# Asymmetric Histone Inheritance Regulates Differential Transcription Re-initiation and Cell Fate Decisions in Mouse Olfactory Horizontal Basal Cells

**DOI:** 10.1101/2025.03.02.641101

**Published:** 2025-03-04

**Authors:** Binbin Ma, Guanghui Yang, Jonathan Yao, Charles Wu, Jean Pinckney Vega, Gabriel Manske, Saher Sue Hammoud, Satrajit Sinha, Abhyudai Singh, Haiqing Zhao, Xin Chen

**Affiliations:** 1 Howard Hughes Medical Institute, Department of Biology, Johns Hopkins University, Baltimore, MD; 2 Department of Biology, Johns Hopkins University, Baltimore, MD; 3 Cellular and Molecular Biology Graduate Program, University of Michigan, Ann Arbor, MI; 4 Department of Human Genetics, University of Michigan, Ann Arbor, MI; 5 Department of Biochemistry, Jacobs School of Medicine and Biomedical Sciences, SUNY at Buffalo, 955 Main St, Buffalo, NY; 6 Electrical & Computer Engineering, University of Delaware, Newark, DE

**Keywords:** Asymmetric histone inheritance, cell fate decision, asymmetric cell division, horizontal basal cell, regeneration, mouse olfactory epithelium

## Abstract

To understand epigenetic inheritance in mammals, we investigate cell division modes and histone inheritance patterns in the mouse olfactory epithelium using an injury-induced regeneration model. Horizontal basal cells (HBCs), the adult stem cells in this tissue, undergo asymmetric division, coinciding with asymmetric histone H4 inheritance *in vivo*. Primary HBCs recapitulate both asymmetric cell division and asymmetric histone inheritance for H4, H3, and H3.3, but not H2A-H2B. Upon mitotic exit, asymmetric histone inheritance correlates with differential enrichment of a key ‘stemness’ transcription factor p63 and asynchronous transcription re-initiation. Single-cell RNA sequencing of paired daughter cells reveals their asymmetric cell fate priming in this multilineage stem cell system. Furthermore, disruption of asymmetric cell division abolishes these asymmetric cellular features, impairing olfactory epithelium regeneration and smell behavior in mice. Together, these findings reveal asymmetric histone inheritance in a mammalian adult stem cell lineage and highlight its biological significance in tissue regeneration and animal behavior.

## INTRODUCTION

In living organisms, maintaining fitness requires the continuous activity of adult stem cells. Proper stem cell function is crucial for healing and growth in response to tissue damage caused by physiological, pathological, or environmental changes. Many types of stem cells divide asymmetrically to balance the maintenance of the stem cell pool with the generation of differentiating cells. Asymmetric cell division is a fundamental mechanism regulating cell fate determination, playing crucial roles in the development, tissue homeostasis and regeneration^1^. However, a long-standing question is how distinct cell fates arise from stem cells that share identical genetic information.

Epigenetics plays a pivotal role in regulating inheritable cellular memory during development as well as tissue maintenance and repair^2^. Histones, the primary carriers of epigenetic information, are critical for regulating gene expression and determining cell fate^3–5^. The nucleosome, the fundamental unit of chromatin, consists of two copies of the canonical core histones—H3, H4, H2A and H2B^6–8^. In addition to their structural roles, histones can undergo extensive post-translational modifications, which are important for their roles in regulating gene expression^9^. The composition, density and position of nucleosomes, as well as distinct histone modifications significantly contribute to cell fate decisions, including self-renewal and differentiation during homeostasis, as well as reprogramming during tissue regeneration^3–5,10^.

Previous studies in *Drosophila* have shown that histones H3 and H4 are inherited asymmetrically during asymmetric divisions of male germline stem cells^11–14^ and female intestinal stem cells^15^. In these adult stem cells, preexisting (old) histones are retained in the stem daughter cell, while newly synthesized (new) histones are enriched in the differentiating daughter cell. In *Drosophila* female germline stem cells^16^ and Wnt3a-induced asymmetrically dividing mouse embryonic stem cells^17^, asymmetric histone patterns are enriched at local genomic regions associated with genes involved in stemness maintenance and differentiation regulation. Recent findings in *Drosophila* male germline stem cells demonstrate that, in addition to old-*versus*-new histone asymmetry, the total amount of histones is asymmetrically distributed between the two daughter cells, indicating differences in nucleosome density between sister chromatids, which is responsible for their differential condensation during mitosis and recruitment of a key DNA replication re-initiation component Cdc6^18^. However, whether asymmetric histone inheritance is conserved in mammalian adult stem cells needs further studies.

To study histone inheritance during cell fate decisions in mammals, we used the mouse olfactory horizontal basal cells (HBCs) located in the olfactory epithelium (OE), a tissue with a life-long ability to regenerate both neuronal and non-neuronal cell types^19^. HBCs have been identified as the *bona fide* adult stem cell population in the OE^20^. HBCs remain quiescent in adult OE under normal physiological conditions. However, in response to acute injury, HBCs are rapidly activated, re-entering cell cycle and regenerating all OE cell types^19,21–23^. Single-cell RNA sequencing has revealed the heterogeneity of the activated HBC pool, with distinct subpopulations giving rise to multiple OE lineages during post-injury repair^24^. HBCs express the transcription factor p63, a master regulator of stem cell proliferative potential and epithelial development^25,26,27,28^. Notably, p63 not only maintains the quiescent state of HBCs but also acts as a molecular switch that triggers their activation upon injury^22,21^. For example, the conditional p63 knockout mice show spontaneous HBC differentiation in uninjured OE and defective HBC self-renewal following injury^23^, underscoring its essential roles in balancing HBC maintenance during homeostasis and activation during regeneration.

In the current study, we combined *in vivo* stem cell tracking, primary HBC culture, mathematical modeling and single-cell transcriptomics to characterize HBC division modes and histone inheritance patterns during OE regeneration. Our results revealed that approximately 40% of HBCs exhibit asymmetric histone inheritance patterns with a corresponding asymmetric p63 distribution during injury-induced OE regeneration. In cultured HBCs, we found that asymmetric histone inheritance influences transcription re-initiation dynamics, transcription factor re-binding, and the specification of distinct cell fates during asymmetric cell divisions (ACDs). Moreover, single-cell (sc) RNA-seq of paired daughter cells derived from single mitosis indicated multi-lineage cell fate priming after one round of ACD. To further understand the biological functions of asymmetric histone inheritance, we used the microtubule depolymerization drug nocodazole (NZ) to abolish ACD in HBCs, which has been shown to effectively disrupt ACD and asymmetric histone inheritance in *Drosophila* male germline stem cells^29^. This treatment led to loss of asymmetric p63 distribution and asynchronous transcription re-initiation in cultured HBCs. Notably, NZ-treated mice exhibited delayed OE regeneration and defective olfactory behaviors, suggesting a functional consequence of this treatment. Our results demonstrate that asymmetric histone inheritance plays a crucial role in regulating differential transcription re-initiation and cell fate decisions in mouse olfactory stem cells during regeneration. These findings highlight the conserved feature of asymmetric histone inheritance and underscore its broader biological significance.

## RESULTS

### Asymmetric p63 Distribution Reflects Asymmetric HBC Division During OE Regeneration

**Figure 1:**
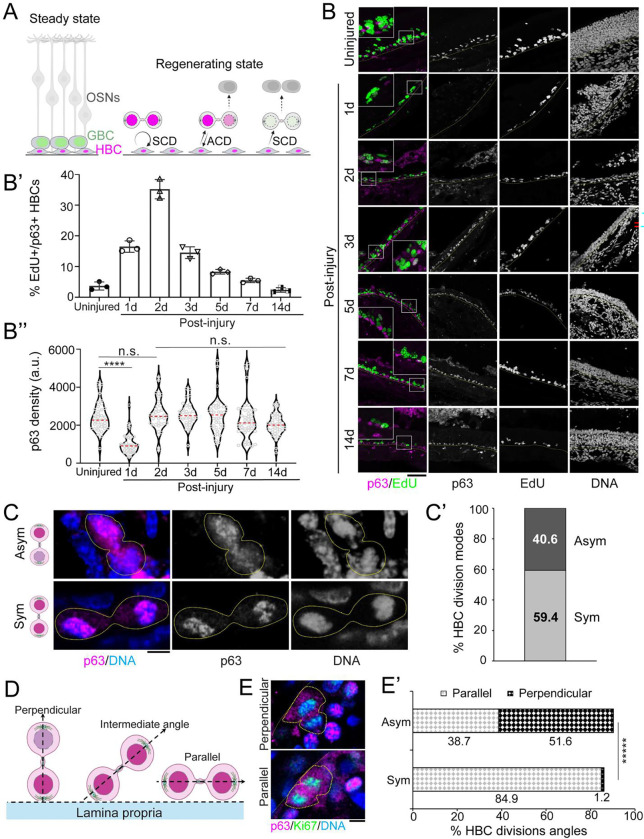
Asymmetric p63 distribution in activated HBCs during OE regeneration. (**A**) A model for asymmetric cell division of HBCs during OE regeneration. In steady state, OE consists of quiescent HBCs, proliferating GBCs and olfactory sensory neurons (OSNs). After acute injury, OE exhibits dramatic cell loss and quickly enters regenerating state: HBCs become activated and symmetrically divide into two self-renew HBCs or two differentiating progenitor cells or asymmetrically divide to one HBC and one transit-amplifying cell. Following that, transit-amplifying cells further generate multilineage OE cell types. (**B**) Post-injury dynamics of EdU labeling (green) in p63+ HBCs (magenta) during OE tissue regeneration. Tissue sections from uninjured and injured olfactory epithelium at 1, 2, 3, 5, 7 and 14 days post-injury. Quantifications of percentage of EdU+ cells (**B’**) and p63 intensities (**B’’**) in p63+ HBCs at different days during OE tissue regeneration. a.u., arbitrary units, n.s., no significance, ****: *P*< 10^−4^, Average (Avg) ± SEM by Mann Whitney test. (**C**) p63 distribution patterns in dividing HBCs. Examples of an anaphase HBC with asymmetric p63 distribution (upper panel) and another with symmetric p63 distribution (lower panel) in an OE section 2 days post-injury. p63 (magenta) and DNA (blue) shown maximum projection. (**C’**) Ratios of asymmetric *versus* symmetric division modes in p63+ HBCs at anaphase/telophase (N=64) 2 days post-injury. (**D**) Illustration of division angle measurements^34^ of anaphase or telophase HBCs. 0–30°: parallel division; 30°−60°: intermediate angle; 60–90°: perpendicular division. (**E**) Representative images of a perpendicular HBC division and a parallel HBC division. p63 (magenta), Ki67 (green), DNA (blue). (**E’**) Quantifications of perpendicular and parallel divisions in asymmetric *versus* symmetric p63+ anaphase and telophase HBCs (N_Asym_=31, N_Sym_=86) Day 2 post-injury. ****: *P*< 10^−4^ by Chi-square test. Cartoon generated by Biorender.com. Scale bars: 50 μm (**B**), 5 μm (**C**) and (**E**).

Under standard laboratory conditions (Steady state), HBCs are largely quiescent in adult mice^30^. The progenitor globose basal cells (GBCs) are responsible for olfactory sensory neuron turnover ^31^. However, following acute injury, such as those causing the loss of sustentacular cells^32^, HBCs become rapidly activated and divide into self-renewing HBCs and differentiating progenitor cells, which further give rise to all OE cell types^19,21^ (Fig. 1A). HBCs can proliferate both symmetrically and asymmetrically, with the latter mode balancing self-renewal and differentiation. It is known that adult stem cells in epithelial systems, such as epidermis and prostate epithelium, undergo ACDs during development^33,34^. However, it remains unclear whether HBCs undergo ACDs during OE regeneration.

To investigate HBC division modes during OE regeneration, we injected 6- to 8-week-old adult mice with methimazole, a drug commonly used to induce OE injury and regeneration in rodents^35–39^. Using a thymidine analog EdU (5-ethynyl 2´-deoxyuridine) labeling, we found a significant increase of p63-expressing HBCs undergoing active DNA replication (p63+/EdU+). The p63+/EdU+ HBCs increased from the uninjured baseline to one day (Day 1 or 1d) post-methimazole treatment, peaking on Day 2 and gradually decreasing thereafter from Day 3 until Day 14 (Fig. 1B, 1B’). Correspondingly, p63 showed a significant decrease on Day 1 post-injury and then quickly recovered from Day 2 to Day 14 (Fig. 1B, 1B’’). These results demonstrate that HBCs undergo dramatic changes in their activation status and cell fate during the early phase of OE regeneration. In line with the EdU pulse results and previous reports^23^, a cell proliferation marker Ki67 showed similar HBC proliferative dynamics, with the peak on Day 2 post-injury (Fig. S1A, S1B). Together, these results demonstrate dramatic HBC activation during the early phase of OE regeneration.

Next, we asked whether ACDs occur during HBC activation at the early stage of OE regeneration. We studied p63 distribution in mitotic HBCs, particularly at telophase, when sister chromatids display clear segregation along with the stem cell fate determinant p63^40,41^. We found that 40.6% of telophase HBCs exhibited asymmetric p63 distribution, while 59.4% displayed symmetric p63 distribution (Fig. 1C, 1C’). Previous studies in the epidermis have revealed that epidermal stem cells orient their spindles in perpendicular to the basement membrane, promoting both asymmetric epidermal stem cell divisions and their step-wise differentiation into cells for stratified epidermis^34^. To determine whether the HBC division angles correlate with the asymmetric outcome of daughter cell fates, we measured the division angles of anaphase and telophase HBCs relative to the basal membrane (Fig. 1D). We found that asymmetrically dividing HBCs preferentially adopted a perpendicular division angle, whereas symmetrically dividing HBCs primarily divided along a parallel angle (Fig. 1E, 1E’).

In summary, these data demonstrate that HBCs undergo ACDs *in vivo* during early OE regeneration, characterized by asymmetric p63 distribution and perpendicular mitotic spindle orientation.

### Asymmetrically Dividing HBCs Exhibit Asymmetric Histone H4 Distribution

**Figure 2:**
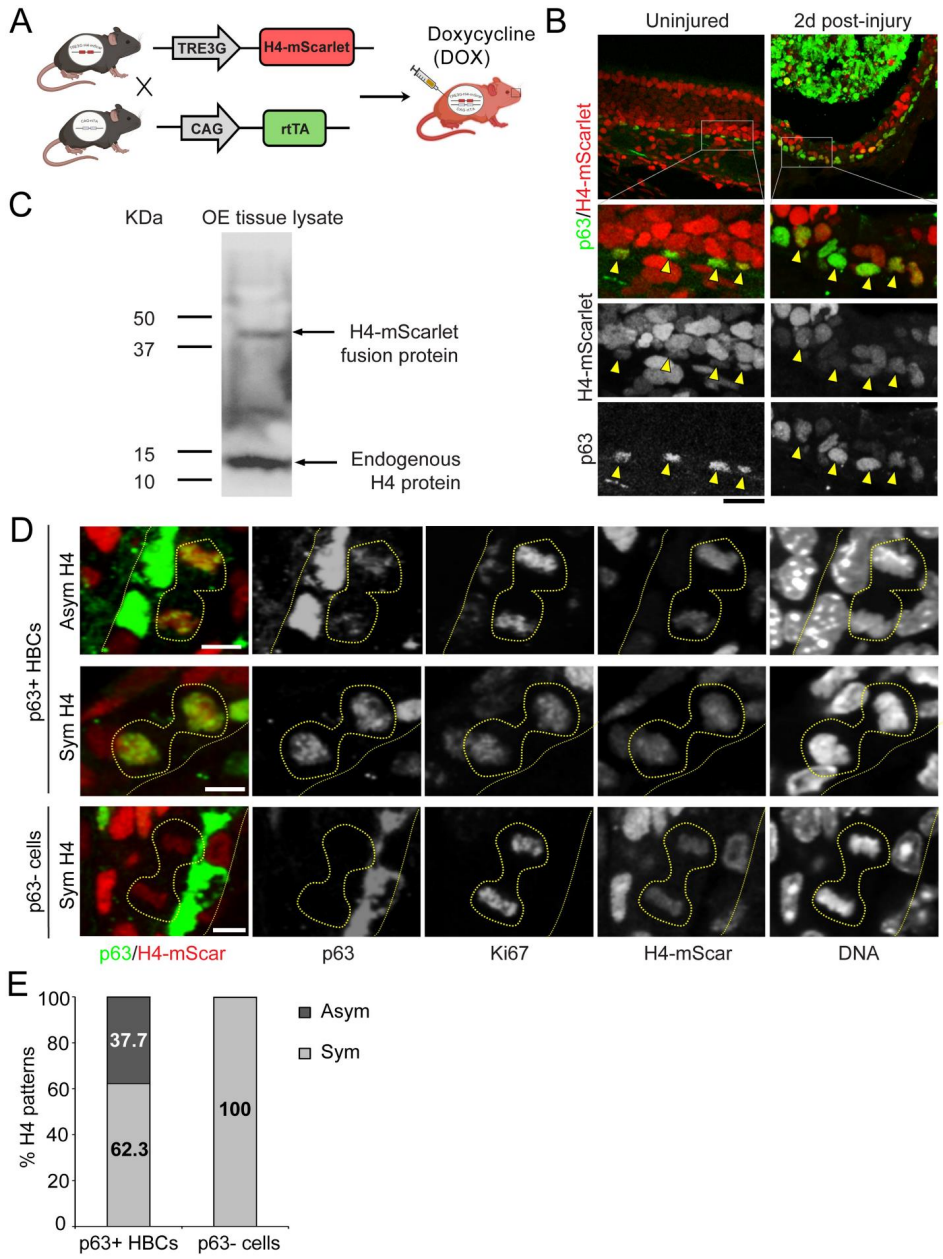
Asymmetric histone H4 distribution in asymmetrically dividing HBCs. (**A**) Illustration of doxycycline-inducible histone H4-mScarlet expression in mice. Cartoon generated by Biorender.com. (**B**) Uninjured and injured OE (2 days post-injury): H4-mScarlet (red), p63 (green) in merged panels. Arrowheads: p63+ HBCs. (**C**) Immunoblot using with anti-H4 antibody on H4mS;rtTA mice OE tissue lysate 2 days post-injury: (top band) H4-mScarlet fusion protein ~39 KD, (bottom band) H4 endogenous protein ~14 KD. (**D**) H4-mScarlet distribution patterns in telophase HBCs. (top) a p63+ HBC with asymmetric H4 distribution, (middle) a p63+ HBC with symmetric H4 distribution, (bottom) a p63- HBC with symmetric H4 distribution, in OE section of H4mS;rtTA mice 2 days post-injury. H4-mScarlet (red), p63 (green) in merged panels. (**E**) Ratios of H4 distribution patterns in p63+ HBCs (N=53) and p63- cells (N=17) at telophase. Scale bars: 50 μm (**B**), 5 μm (**D**).

To investigate histone distribution patterns in asymmetrically dividing HBCs, we first focused on histone H4 because it has no known variants among all canonical histones^42–44^ therefore its level can faithfully reflect the total nucleosome amount genome-wide. To directly visualize H4 distribution patterns, we used a Tet-inducible histone transgenic mouse model. Mice carrying a tetracycline-responsive element (TRE3G)-driven H4-mScarlet (H4mS) were crossed with CAG-rtTA mice, allowing for the induction of H4-mScarlet expression upon doxycycline (DOX) injection in H4mS;rtTA progenies (Fig. 2A).

We first examined H4 expression patterns in uninjured and injured OE (Day 2 post-injury). We found that H4-mScarlet labeled all olfactory cell types, including p63+ HBCs (Fig. 2B). To access the relative expression levels of the H4-mScarlet fusion protein with the endogenous H4, we performed immunoblot experiments using OE tissues and found that the fusion protein accounts for approximately 10% of endogenous H4, indicating that endogenous H4 remains abundant and predominant in H4mS;rtTA OE cells (Fig. 2C). Although H4-mScarlet transgene brings only a small addition to the total H4 amount, it allows for visualizing H4 distribution patterns *in vivo*. We further confirmed the dynamics of OE recovery post-injury between the control and H4mS;rtTA mice (Fig. S2A). Compared to the controls, H4mS;rtTA OE cells exhibited robust H4-mScarlet expression without significant differences in OE thickness or the ratio of Ki67+ HBCs during early OE regeneration (Fig. S2B, S2C). Together, these results indicate that the Tet-inducible histone transgenic mouse line expresses H4-mScarlet fusion proteins without compromising OE homeostasis or regeneration.

To investigate whether histone distribution patterns exhibit asymmetry during ACD of HBCs, we analyzed the injured OE from H4mS;rtTA mice (Day 2 post-injury), when HBC proliferation is at the peak. Notably, we found that histone H4 levels were unequal in asymmetric p63+ HBCs at telophase, with higher H4 levels correlating with increased p63 abundance. In contrast, symmetric p63+ HBCs displayed equal histone H4 distribution at telophase. Additionally, H4 showed symmetric distribution in 100% p63-negative cells of regenerating OE (Fig. 2D, 2E). Quantification of both H4 and p63 levels revealed that 37.7% of telophase HBCs displayed asymmetric H4 and p63 distribution, while 62.3% exhibited symmetric patterns of both H4 and p63 (Fig. 2D, 2E).

Together, these results suggest that the asymmetric inheritance of histone H4 is closely associated with the asymmetric division of HBCs during early OE regeneration, suggesting a link between stem cell division asymmetry and histone inheritance asymmetry.

### Asymmetrically Dividing HBCs Exhibit Symmetric Histone H2A-H2B Distribution

Canonical histone proteins are mainly synthesized and incorporated into DNA during replication as an octamer structure, consisting of two copies each of H3, H4, H2A, and H2B^6–8^. It has been shown that (H3-H4)_2_ tetramers, deposited during DNA replication, remain unsplit, while H2A and H2B are incorporated as dimers^45–49^. Additionally, throughout the cell cycle H2A and H2B are more dynamically associated with DNA compared to the more stable (H3-H4)_2_ tetramers^50^. To determine whether the detected asymmetric histone patterns are specific to H4, we examined the distribution of H2A-H2B dimers using nanobody staining in the OE (Fig. S3A). We first performed nanobody staining in uninjured OE and found ubiquitous H2A-H2B signals in all OE cell types, including p63+ HBCs (Fig. S3B). This confirms that the H2A-H2B nanobody efficiently labels histone H2A-H2B dimers in the OE. We next characterized H2A-H2B patterns in injured OE (Day 2 post-injury) and found that both p63+ HBCs and early differentiated cells were positively stained by the H2A-H2B nanobody (Fig. S3B).

Next, we focused on mitotic HBCs, particularly those in telophase, to study how H2A-H2B signals are segregated in relation to p63 distribution patterns. The levels of H2A-H2B in the two sets of sister chromatids were nearly equal in both symmetrically dividing HBCs with similar p63 levels and asymmetrically dividing HBCs with different p63 levels (Fig. S3C, S3D), suggesting that H2A-H2B levels are independent of p63 distribution patterns and HBC division modes.

Together, these results demonstrate the symmetric inheritance of histone H2A-H2B in both symmetrically and asymmetrically dividing HBCs during early OE regeneration.

### Primary Cultured HBCs Undergo Asymmetric Division and Display Asymmetric Histone Inheritance

**Figure 3:**
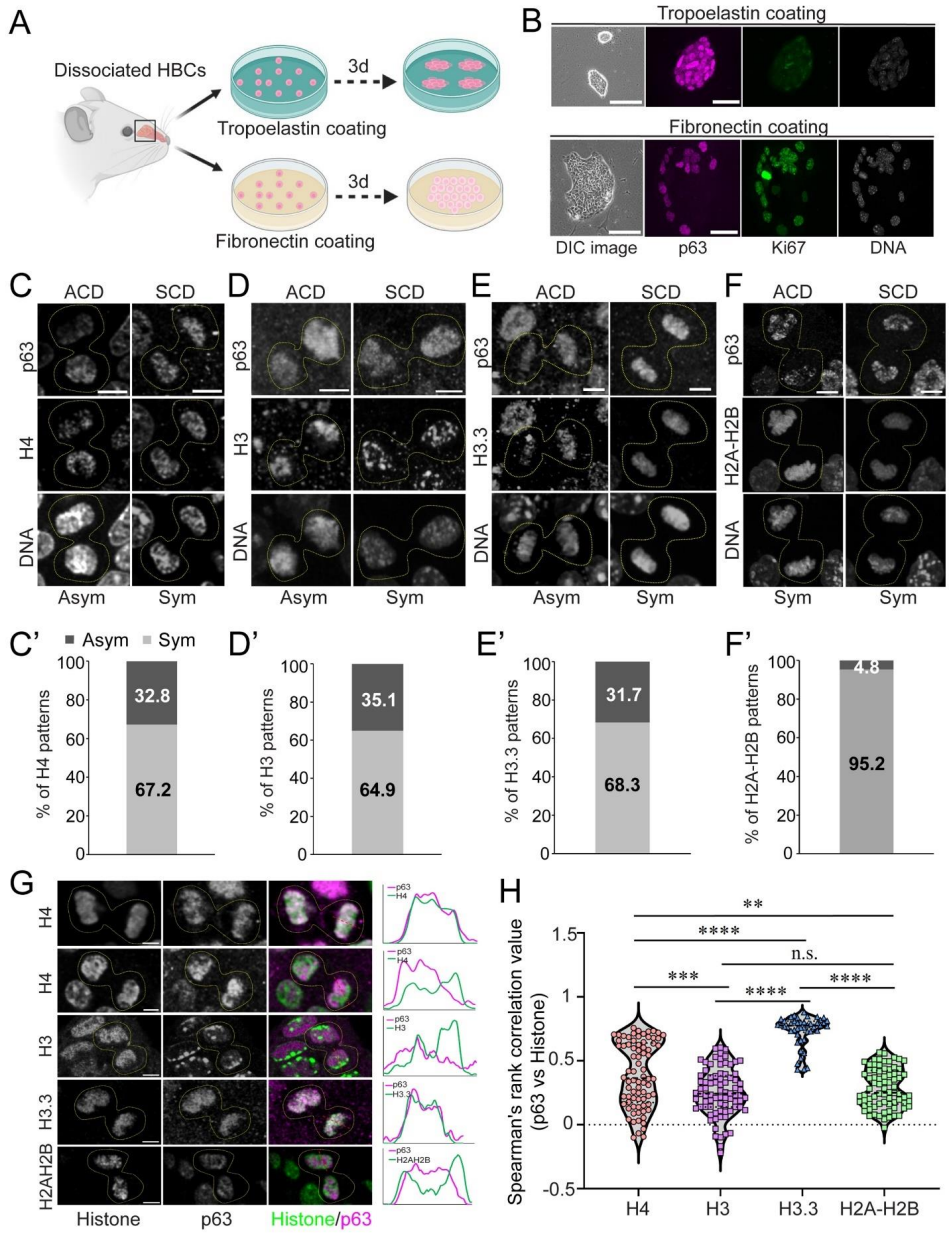
Asymmetric division and histone inheritance in primary cultured HBCs. (**A**) Illustration of primary HBCs culture with different extracellular matrix coating conditions to study stem cell division modes and histone inheritance. Tropoelastin maintains less proliferation of HBCs, while Fibronectin promotes proliferation of activated HBCs. Cartoon generated by Biorender.com. (**B**) The comparison of primary cultured HBCs on Tropoelastin-coated and Fibronectin-coated dish with bright field of stem cell colonies, stem cell marker p63 (magenta) and proliferation marker Ki67 (green). (**C**) The symmetrically and asymmetrically dividing HBCs (N=64) in the colony with p63, H4 and DNA staining. (C’) Quantification showed the percentage of H4 patterns between sister chromatids of p63+ telophase HBCs: Asymmetric H4=32.8%, Symmetric H4=67.2%. (**D**) The symmetrically and asymmetrically dividing HBCs (N=74) in the colony with p63, H3 and DNA staining. (D’) Quantification showed the percentage of H3 patterns between sister chromatids of p63+ telophase HBCs: Asymmetric H3=35.1%, Symmetric H3=64.9%. (**E**) The symmetrically and asymmetrically dividing HBCs (N=39) in the colony with p63, H3.3-mScarlet and DNA staining. (E’) Quantification showed the percentage of H3.3 patterns between sister chromatids of p63+ telophase HBCs: Asymmetric H3.3=31.7%, Symmetric H3.3=68.3%. (**F**) The symmetrically and asymmetrically dividing HBCs (N=42) in the colony with p63, H2A-H2B and DNA staining. (F’) Quantification showed the percentage of H2A-H2B patterns between sister chromatids of p63+ telophase HBCs: Asymmetric H2A-H2B =4.8%, Symmetric H2A-H2B=95.2%. (**G**) Representative images and line plots showing the colocalization pattern of histone H4, H3, H3.3 and H2A-H2B (green) with p63 (magenta) of cultured telophase HBCs from injured OE (2 days post-injury). (**H**) The colocalization analysis of stem cell marker p63 with different histones, including H4, H3, H3.3 and H2A-H2B. Average colocalization indexes are H4: 0.39±0.03; H3: 0.26±0.02; H3.3: 0.74±0.02; H2A-H2B: 0.28±0.02. Values represent average ± SEM. n.s., no significance, ** p < 0.01, *** p < 0.001, **** p< 0.0001 by Mann Whitney test. Scale bars: 100 μm (**B**-brightfield); 20 μm (**B**-immunostaining); 5 μm (**C**), (**D**), (**E**), (**F**) and (**G**). ACD, asymmetric cell division; SCD, symmetric cell division. ACD vs. SCD is based on p63; Asym vs. Sym is based on histones for panels (**C**), (**D**), (**E**), (**F**).

Primary cultured HBCs have previously been used as a model system to recapitulate *in vivo* HBC behavior and activity^51^, enabling greater flexibility for manipulation and single-cell tracking. To better modulate HBC activation, we further optimized the extracellular matrix coating to facilitate studies on division mode and epigenetic regulation during HBC activation at single-cell resolution (Fig. 3A). Specifically, using Tropoelastin coating, cultured HBCs can be maintained in a quiescent state, forming compact, small colonies characterized by high p63 and low Ki67 levels (Fig. 3B). However, switching to Fibronectin coating induced colony expansion and cell flattening, accompanied by p63 downregulation and a drastic upregulation of Ki67, indicating a transition from a dormant state to an activated state (Fig. 3B). Thus, this manipulation effectively recapitulates HBC activation post-injury, establishing it as an *ex vivo* system that complements and extends *in vivo* studies of HBCs.

We performed p63 and histone antibody staining to confirm that ACD and asymmetric histone inheritance detected in HBCs post-injury *in vivo* can be recapitulated *ex vivo*. First, we observed similar ratios of asymmetric H4 inheritance accompanied by asymmetric p63 distribution in telophase HBCs *ex vivo* (Fig. 3C, 3C’), compared to HBCs post-injury *in vivo* (Fig. 2E). Similar asymmetric inheritance patterns of H3 were observed (Fig. 3D, 3D’). Notably, the histone variant H3.3 also displayed asymmetric inheritance patterns in primary cultured HBCs (Fig. 3E, 3E’). Consistent with the *in vivo* results (Fig. S3C, S3D), histone H2A-H2B displayed a symmetric inheritance pattern, regardless of p63 distribution between sister chromatids (Fig. 3F, 3F’). To validate our immunostaining results in fixed samples, we performed live imaging of cultured HBCs expressing H4-Dendra2, demonstrating asymmetric H4 segregation at telophase (Fig. S4). Together, primary cultured HBCs exhibited asymmetric histone inheritance during ACDs, consistent with the *in vivo* results shown in Figure 1, 2 and S3.

Next, to quantitatively assess the molecular association between p63 and different histones, we measured relative Pearson colocalization coefficients^16,52,53^ using high-spatial-resolution microscopy images, where higher coefficients indicate colocalization and lower coefficients indicates separation between two signals. We observed varying degrees of colocalization between p63 and different histone proteins in telophase HBCs, a stage when p63 rebinds to chromosomes as they decondense when entering the next interphase (Fig. 3G). Notably, the histone variant H3.3, which is closely associated with transcriptional activation and enriched in open chromatin regions, exhibited a high degree of colocalization with p63 in telophase HBCs (Fig. 3G, 3H). In contrast, canonical histones H3 and H2A-H2B exhibited significantly lower colocalization with p63 compared to H3.3, likely due to their abundance in more closed chromatin regions (Fig. 3G, 3H). Intriguingly, histone H4 exhibited a bimodal colocalization pattern with p63 (Fig. 3G, 3H), likely due to the absence of an H4 variant^42–44^. Therefore, the overall colocalization pattern between p63 and H4 likely reflects p63 association with (H3.3-H4)₂ and separation from (H3-H4)₂ (Fig. 3H).

Together, our results suggest that the histone tetramer (H3/H3.3-H4)₂ may serve as a crucial epigenetic information carrier in olfactory HBCs.

### Primary Cultured HBCs Exhibit Asynchronous Cell Cycle Progression

Stem cell self-renewal and differentiation lead to the specification of distinct cell fates. This process is essential for tissue and organ growth, homeostasis, and regeneration, and is closely linked to cell cycle progression^54^.

To directly visualize and precisely distinguish cell cycle stages in primary cultured HBCs, we utilized the engineered Fluorescence Ubiquitin Cell Cycle Indicator (FUCCI-CA5) reporter system. This systems employs a CUL4^Ddb1^-sensitive hCdt1-based probe and an APC^Cdh1^-sensitive hGem-based probe^55^, allowing clear distinguishment of interphase boundaries between G1, S, and G2 phases using distinct fluorescent markers (Fig. S5A). Under Fibronectin-coated culture conditions, activated HBCs transfected with FUCCI-CA5 displayed red (G1 phase), green (S phase), and yellow (G2 phases) fluorescence (Fig. S5B), confirming that the FUCCI reporter reliably indicates distinct cell cycle stages in HBCs.

Using long-term live-cell imaging, we found that daughter cells derived from a single HBC division exhibited asynchronous progression from G1 to S phase, with one transitioning from G1 (red) to S (green) phase before the other. This asynchrony persisted as the daughter cells entered the subsequent G2-M phase (Fig. S5C).

To explore potential mechanisms underlying asynchronous cell cycle progression, we co-labeled the FUCCI system with histone H4 staining and found that this asynchrony was associated with the prior asymmetric distribution of histone H4 between sister chromatids during M phase (Fig. S5D, S5E). These results suggest that H4 asymmetry may contribute to the asynchronous cell cycle progression of daughter cells derived from HBC asymmetric division, consistent with previous findings in *Drosophila* male germline stem cells^56^.

Using live-cell imaging with the FUCCI-CA5 reporter, we analyzed cell cycle length of primary HBCs cultured on Tropoelastin-coated, or under short-term and long-term Fibronectin-coated conditions (Fig. S5F). Consistent with the finding that Tropoelastin maintains HBCs in a dormant state while Fibronectin induces their proliferation (Fig. 3A, 3B), the average cell cycle length decreased significantly from 38 hours on Tropoelastin-coated condition to 14 hours on Fibronectin-coated condition (Fig. S5F). Notably, the G1 phase of activated HBCs was significantly shortened under Fibronectin-coated conditions (Fig. S5F). To further explore the relationship between cell cycle changes and HBC activation, we developed a mathematical model. Remarkably, our simulations accurately recapitulated the *in vivo* EdU labeling data over time (Fig. S5G; Fig. 1B, 1B’).

In summary, these findings demonstrate that primary cultured HBCs under Tropoelastin- and Fibronectin-coated conditions reliably mimic distinct stem cell features *in vivo*, making them a suitable model for studying stem cell division modes, histone inheritance patterns, and their biological consequences.

### Asymmetrically Dividing HBCs Display Asynchronous Transcription Re-initiation

**Figure 4:**
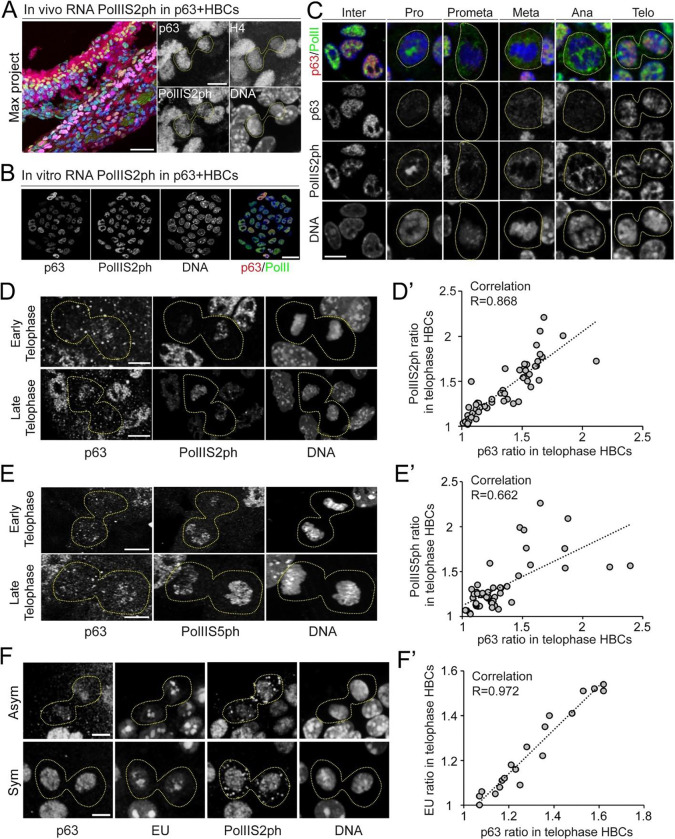
Asynchronous transcription re-initiation in asymmetrically dividing HBCs. (**A**) Asymmetric RNA PolIIS2ph distribution with staining of p63 (magenta), PolIIS2ph (green), H4-mScalet (red) and DNA (blue) during asymmetric cell divisions of p63+ telophase HBCs from injured OE (2 days post-injury). (**B**) RNA PolIIS2ph distribution with staining of p63 (red), PolIIS2ph (green) and DNA (blue) in the primary cultured HBCs. (**C**) Dynamics of RNA PolIIS2ph distribution with staining of p63 (red), PolIIS2ph (green) and DNA (blue) during mitosis. (**D**) Asymmetric RNA PolIIS2ph distribution with staining of p63, PolIIS2ph and DNA during asymmetric cell divisions of early and late telophase HBCs (N=55) from primary culture. D’. Correlation analysis of p63 ratios and PolIIS2ph ratios between two sister chromatids in p63+ telophase HBCs. Correlation coefficient R=0.868. (**E**) Asymmetric PolIIS5ph distribution with staining of p63, PolIIS5ph and DNA during asymmetric cell divisions of early and late telophase HBCs (N=50). E’. Correlation analysis of p63 ratios and PolIIS5ph ratios between two sister chromatids in p63+ telophase HBCs. Correlation efficient R=0.662. (**F**) Asymmetric and symmetric EU distribution with staining of p63, PolIIS2ph, EU and DNA in telophase HBCs (N=19) from primary culture. F’. Correlation analysis of p63 ratios and EU ratios between two sister chromatids in p63+ telophase HBCs. Correlation efficient R=0.972. Scale bars: 50 μm (**A**-section) and 5 μm (**A**-telophase HBCs); 20 μm (**B**); 10 μm (**C**); 5 μm (**D**), (**E**) and (**F**).

The histone variant H3.3 is closely linked to chromatin accessibility and active transcription in euchromatin^57^. To assess transcriptional activity during asymmetric HBC divisions, we examined RNA polymerase II phosphorylated at Serine 2 (Pol IIS2ph), a marker of active transcription elongation^58^, in co-labeling with p63 on OE sections expressing H4-mScarlet. In telophase HBCs, we observed an asymmetric pattern of Pol IIS2ph signals that correlate with asymmetric p63 distribution and histone H4 asymmetry (Fig. 4A). These *in vivo* results indicate that transcription re-initiates asynchronously, potentially regulated by nucleosome asymmetry between sister chromatids as chromatin reopens upon mitotic exit.

To further investigate the dynamics of transcription re-initiation, we used the primary cultured HBCs, which yielded more telophase HBCs for quantitative analysis (Fig. 4B). In p63+ mitotic HBCs, we tracked the dynamic localization of Pol IIS2ph and noted that its signal was diffusely distributed throughout the cell from prometaphase to anaphase but re-bound to de-condensing chromosomes in late telophase (Fig. 4C). Consistent with *in vivo* observations (Fig. 4A), Pol IIS2ph showed an asymmetric distribution in p63+ HBCs at telophase (Fig. 4D), with quantification confirming a positive correlation between p63 and Pol IIS2ph (Fig. 4D’). To assess whether this asymmetry is specific to Pol IIS2ph, we examined RNA polymerase II phosphorylated at Serine 5 (Pol IIS5ph), a marker of transcription initiation at promoter regions^59–61^. Similar to Pol IIS2ph, Pol IIS5ph exhibited asymmetric distribution, aligning with p63 asymmetry in p63+ HBCs at telophase (Fig. 4E, 4E’).

Next, to further explore the mechanisms underlying RNA Pol II asymmetry, we developed a mathematical model to simulate the ratio of RNA Pol II intensity between sister chromatids in telophase HBCs. The model assumes that in a fraction of single cells undergoing ACD, RNA Pol II is asymmetrically partitioned based on its chromatin binding affinity. Fitting the model-predicted RNA Pol II intensity ratios to experimental data revealed a 60% difference in RNA Pol II binding affinity between sister chromatids. This differential affinity may drive transcription re-initiation asymmetry, potentially influencing cell fate determination during early-stage HBC regeneration *in vivo* (Fig. S6).

To directly monitor transcriptional activity, we used 5-ethynyluridine (EU) to label nascent RNA in cultured HBCs. We found that nascent RNA synthesis also exhibited an asymmetric pattern, correlating positively with the asymmetric distribution of p63 and Pol IIS2ph in telophase p63+ HBCs (Fig. 4F, 4F’).

Together, these results suggest that transcription is differentially re-initiated in telophase HBCs and that this asymmetry may be driven by asymmetric histone inheritance, contributing to distinct daughter cell fates following stem cell ACD.

### scRNA-seq of Paired Daughter HBCs Identifies Asymmetric Cell Fate Priming

**Figure 5:**
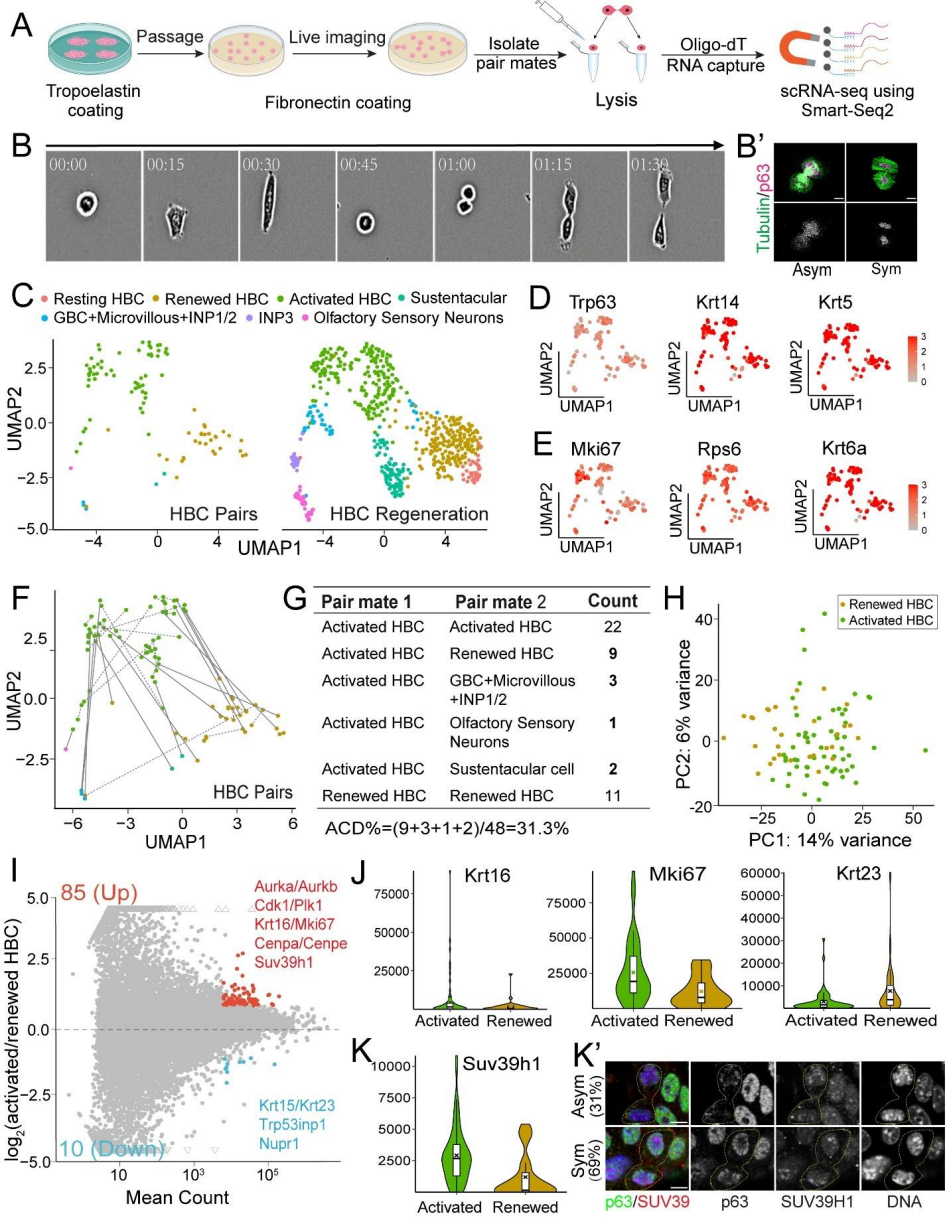
Asymmetric cell fate priming of activated HBCs by paired daughter cell RNA sequencing. (**A**) Scheme of paired daughter cell preparation for the scRNA-seq experiment. Primary cultured HBCs isolated from injured OE (2 days post-injury) were used for the paired daughter cell scRNA-seq. Cartoon generated by Biorender.com. (**B**) Live cell imaging of cultured individual HBC to track the division from one mother cell to two daughter cells. (**B’**) The HBCs fate and pair information was confirmed by staining of p63 (magenta) and tubulin (green) after division. (**C**) Clustering of HBCs paired cells after integrating the scRNA-seq data (GSE286046) into published dataset (GSE99251 and GSE95601). (**D**) Feature plots showing expression of HBCs marker genes *Trp63*, *Krt14* and *Krt5* in collected single cells. (**E**) Feature plots showing expression of cell cycle and wound response genes *Mki67*, *Rps6* and *Krt6a* of collected single cells. (**F**) Paired information of all single cells examined. Solid lines indicate asymmetric pairs cross different clusters. Dotted lines indicate symmetric pairs in the same clusters. (**G**) Statistics of paired information showing symmetric and asymmetric divisions in F: 31.3% (15/48) of HBC pairs show asymmetric divisions. (**H**) PCA plot using scRNA-seq results of all renewed and activated HBC cells. (**I**) MA-plot shows differentially expressed genes between renewed and activated HBC cells. Genes highly expressed in activated HBC cells are labeled red with total gene number indicated (85 Up), for example the cell cycle and proliferation associated genes (*Aurka*, *Aurkb*, *Cdk1*, *Plk1*, *Mki67*, *Cenpa* and *Cenpe*). Genes showing lower expression in activated HBC cells are labeled blue (10 Down), for example the renewed HBC marker genes (*Krt15* and *Krt23*) and transcriptional regulator (*Nupr1*). (**J**) Expression of proliferation marker gene (*Mki67*) and newly identified activated HBCs marker (*Krt16*) and renewed HBCs marker (*Krt23*) in activated and renewed HBC cells. (**K**) Expression of histone methyltransferase gene Suv39h1 in activated and renewed HBC cells. Gene counts normalized by external ERCC spike-in are used in the violin and box plots. Average expression of genes examined in different groups of cells are labeled with the ‘x’ symbol. (**K’**) Asymmetric and symmetric SUV39H1 distribution with antibody staining of p63 (green), SUV39H1 (red) and DNA (blue) in p63+ telophase HBCs (N=29) from primary culture. Scale bars: 5 μm (**B**’) and (**K**’).

To dissect the molecular features of asymmetrically dividing HBC daughters with asymmetric histone inheritance, we profiled their single-cell transcriptomes using the G&T-seq (Genome & Transcriptome-sequencing) method^62^. To effectively isolate paired daughter cells, we optimized the primary culture system, enabling robust HBC attachment and division (Fig. 5A). Live-cell imaging was used to track the entire division process, allowing precise localization of paired daughters after a single HBC division, which was further validated with p63 and Tubulin immunostaining (Fig. 5B).

In this experiment, we collected 48 paired daughter cells for single-cell RNA sequencing (scRNA-seq). By comparing our results to published scRNA-seq datasets^24,63^, we found that most pairs could be assigned to clusters corresponding to renewed (less activated) or activated HBCs (Fig. 5C, S7A). Consistent with this, all 96 cells expressed the HBC marker *Trp63* (encoding p63), as well as *Krt14* and *Krt5* (Fig. 5D). Additionally, the majority of cells expressed wound-response and cell cycle genes, such as *Mki67*, *Rps6*, and *Krt6a*, which are characteristic of HBC activation (Fig. 5E). However, markers for other OE cell types were absent, including progenitor GBC markers (*Ascl1*, *Kit*, *Lgr5*), sustentacular cell marker (*Il33*), and mature olfactory sensor neuron markers (*Gng13*, *Omp*) (Fig. S7B, S7C). These findings suggest that, although the collected cells have acquired an activated state, they remained stem cell-like.

Using the cluster information from published datasets, we identified both symmetric and asymmetric daughter cell pairs (Fig. 5F). Among the 48 pairs, 22 consisted of two activated HBC daughter cells, and 11 consisted of two renewed HBC daughter cells, indicating symmetric divisions. Notably, 15 pairs (31.3%) exhibited asymmetric fates, including nine Activated HBC:Renewed HBC pairs, three Activated HBC:GBCs/MV/INP pairs, two Activated HBC:Sustentacular cells pairs, and one Activated HBC:OSN pair (Fig. 5G). These asymmetric pairs highlight the multilineage cell fate priming that occurs after a single round of stem cell division during HBC activation and early differentiation.

Principal Component Analysis (PCA) revealed transcriptomic diversity and a population shift between renewed and activated HBCs in the paired daughter cells (Fig. 5H). Differential gene expression analysis identified 85 upregulated genes and 10 downregulated genes in activated HBCs compared to renewed HBCs (Fig. 5I). The genes enriched in activated HBCs were significantly associated with Gene Ontology (GO) terms related to ‘cell division’, ‘cell cycle’, ‘mitotic cytokinesis’, ‘chromosome segregation’, and ‘mitotic sister chromatid segregation’ (Fig. S7D), suggesting that the transition from renewed to activated states may involve mitotic events and cell cycle progression during early OE regeneration. Among the upregulated genes in activated HBCs, cell cycle genes *Mki67*, *Cdk1*, *Cdc20*, and *Ccnb1* (*cyclin B1*), along with HBC marker genes (*Krt5*, *Trp63, Krt14, Krt16*, and *Krt23*), could serve as markers of ACD that produces Activated HBC:Renewed HBCs pairs (Fig. 5J, S7E). Intriguingly, we found that the transcript levels of histone methyltransferase SUV39H1 may play a role in HBC cell fate determination, as confirmed by immunostaining (Fig. 5K, 5K’). Notably, we also found higher levels of histone *H3.3* transcripts in activated HBC (Fig. S7F), which is consistent with the distribution patterns of H3.3 in primary culture HBCs (Fig. 3E, 3E’).

Together, paired daughter cell scRNA-seq revealed asymmetric cell fate priming and identified candidate markers for studying ACD during HBC activation and early differentiation.

### Perturbing Asymmetric Histone Inheritance Impairs Asynchronous Transcription Re-initiation

In *Drosophila*, asymmetric microtubule activities facilitate the polarized attachment of sister chromatids, allowing for asymmetric histone inheritance and differential cell fate decisions in male germline stem cells. Treatment with Nocodazole (NZ), a reversible microtubule polymerization inhibitor, disrupted this process, leading to randomized histone inheritance and impaired cell fate determination^14^.

Using live-cell imaging of microtubule activity and *p63* transcription via a *p63*-EGFP reporter^64^, we found that approximately 31% of dividing HBCs displayed asymmetric *p63*-EGFP levels and temporarily asymmetric microtubule dynamics, while 69% exhibited symmetric *p63*-EGFP and microtubule dynamics (Fig. S8A, S8B). These live-imaging results are consistent with p63 protein distribution patterns observed *in vivo* (Fig. 1C, 1C’), indicating the potential role of microtubule dynamics in regulating HBC division modes.

**Figure 6:**
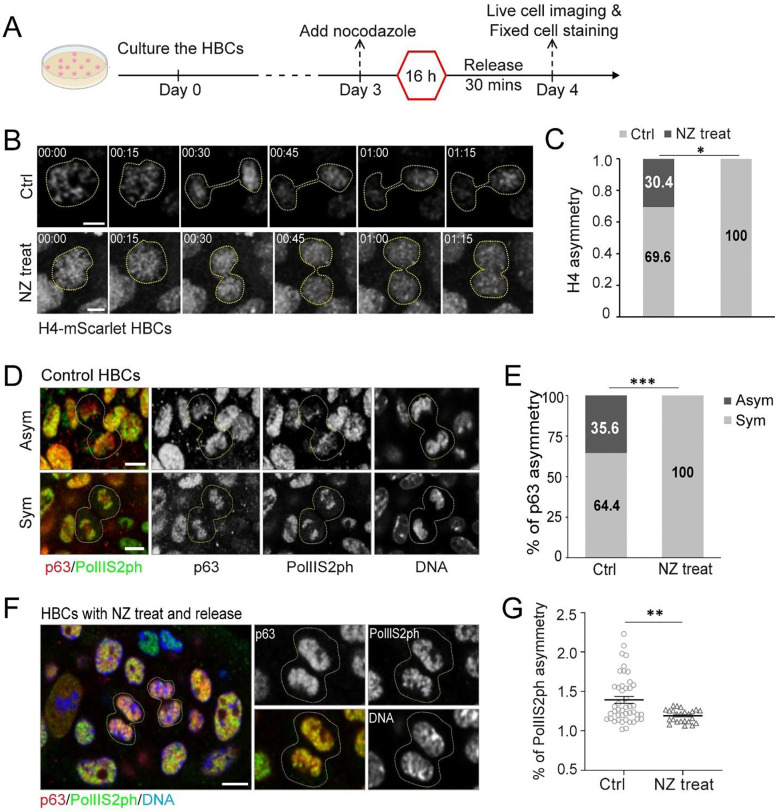
Impaired asynchronous transcription re-initiation by disruption of the asymmetric histone inheritance. (**A**) Illustration of NZ treatment in primary culture HBCs. (**B**) Live cell imaging showing the readout of NZ treatment in primary culture H4-mScarlet HBCs. (**C**) Quantification of histone H4 asymmetry from live imaging of H4-mScarlet HBCs with and without NZ treatment. * P<0.05 by Chi-squared test. (**D**) The symmetrically and asymmetrically dividing HBCs in control group without NZ treatment with staining for p63 (red), RNA Pol IIS2ph (green) and DNA dye Hoechst 33342 (grey). (**E**) Quantification for the percentage of p63 asymmetry in p63+ telophase HBCs (N_Ctrl_=45; N_NZ treat_=24). *** P<0.001 by Chi-squared test. (**F**) The symmetrically dividing HBCs in telophase HBCs with NZ treatment and release with staining for p63 (red), RNA Pol IIS2ph (green) and DNA dye Hoechst 33342 (grey). (**G**) Quantification for the percentage of RNA Pol IIS2ph asymmetry in p63+ telophase HBCs (N_Ctrl_=45; N_NZ treat_=24). ** P<0.01 by Mann Whitney test. Scale bars: 5 μm (**B**), (**D**) and (**F**).

To access the acute response to NZ treatment, we cultured HBCs in the presence of NZ (Fig. S7C). After 16 hours, most HBCs were arrested at prometaphase. Upon NZ washout, HBCs re-entered mitosis in a time-dependent manner, with the majority reaching telophase 30 minutes post-release (Fig. S8C). Under these conditions, live-cell imaging revealed that 100% of HBCs exhibited symmetric H4 segregation (Fig. 6A–C), in contrast to untreated controls, where a substantial proportion (30.4%) displayed asymmetric H4 segregation (Fig. 6B, 6C).

To investigate the consequences of losing asymmetric H4 distribution, we examined p63 and Pol IIS2ph distribution patterns in NZ-treated HBCs. Immunostaining of p63 revealed a significant reduction in asymmetric division mode in treated cells (Fig. 6D–F). Additionally, NZ-treated HBCs showed a significant decrease in asymmetric Pol IIS2ph distribution (Fig. 6D, 6F–G).

Together, these findings demonstrate that disrupting histone H4 asymmetry through manipulating microtubule activities abolishes asymmetric HBC division and impairs differential transcription re-initiation in cultured primary cells.

### Disrupting Asymmetric Histone Inheritance Delays OE Regeneration and Alters Smell Behavior

**Figure 7:**
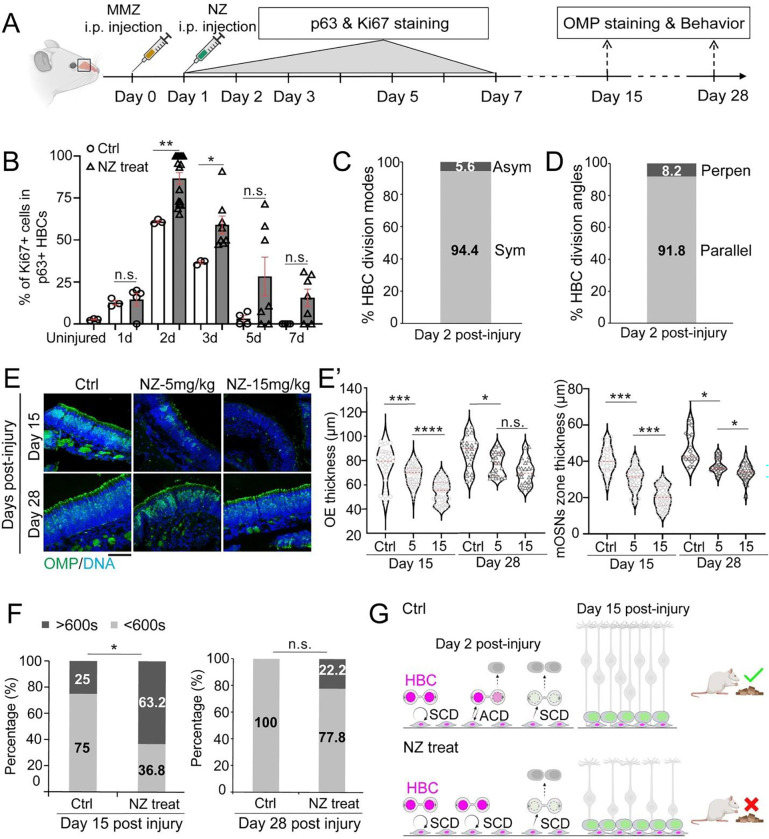
Delayed OE regeneration and impaired olfactory behavior by disruption of the asymmetric histone inheritance. (**A**) Illustration for functional experimental design with Methimazole (MMZ) injection (Day 0) and Nocodazole (NZ) injection (Day 1) in 6–8-week adult mice. p63 and Ki67 staining were performed in early regeneration stage (Day 1-Day 7). OMP staining and food seeking behavior was performed in the late regeneration stage (Day 15 and Day 28). (**B**) Percentage of Ki67+ cells in p63+ HBCs from 2 days post-injury OE with NZ injection dosage of 15 mg/kg, in comparison to the OE at 2 days post-injury without NZ (Figure S1 A, B). * P<0.05, ** P<0.01, n.s., not significant by Mann Whitney test. (**C**) Ratios of asymmetric *versus* symmetric division modes in p63+ HBCs at anaphase/telophase (N=36) from NZ treated OE at 2 days post-injury. (**D**) Quantifications of parallel and perpendicular divisions in p63+ anaphase and telophase HBCs (N=49) from NZ treated OE at 2 days post-injury. (**E**) The OE staining with mature OSN marker OMP (green) on Day 15 and Day 28 post-injury with NZ injection dosage of 5mg/kg and 15 mg/kg. (**E’**) Quantification for the thickness of whole OE and OMP+ mOSN zone in Day 15 and Day 28 post-injury with NZ injection dosage of 5mg/kg and 15 mg/kg. * P<0.05, *** P<0.001, *** P<0.0001, n.s., not significant by Mann Whitney test. (**F**) Mice food seeking behavior results from 15 days’ (NCtrl=16; NNZ treat=19) and 28 days’ (NCtrl=7; NNZ treat=9) OE recovery with NZ injection dosage of 15 mg/kg. * P<0.05, n.s., not significant by Chi-square test. (**G**) Cartoons depicting the predicted recovery of NZ treated mice in response to Methimazole-induced damage. Under normal conditions, the activated HBCs perform both symmetric cell divisions and asymmetric cell divisions in early regeneration stage, then substantial OMP+ mature OSNs are generated by Day 15 post-injury, which enable the mice to recover the olfaction ability. While in NZ treated conditions, the activated HBCs only perform symmetric cell divisions in early regeneration stage, which delays the OE regeneration and impairs the olfactory behavior. Cartoon generated by Biorender.com.

To investigate the *in vivo* consequences of losing asymmetric histone inheritance, we administered NZ injections to OE-injured mice and analyzed the regeneration process. During early regeneration, proliferative activity of HBCs was significantly higher at Days 2 and 3 post-injury (Fig. 7B), suggesting that NZ treatment promoted proliferation by increasing symmetric divisions of activated HBCs. Additionally, dividing HBCs predominantly exhibited symmetric p63 distribution and parallel division angles at Day 2 post-injury (Fig. 7C, 7D). These results indicate that NZ treatment disrupted the balance between asymmetric and symmetric divisions during early OE regeneration.

To examine the effect of early-stage disruption of HBC asymmetric division on OE regeneration, we further collected OE tissue on Day 15 and Day 28 post-injury (Fig. 7A, 7E). At Day 15, non-NZ-treated control mice showed substantial recovery in the OE thickness and the amount of OMP+ mature OSNs (Fig. 7E, 7E’). In contrast, NZ-treated mice exhibited a significantly thinner OE with thinner OMP+ mature OSN zone (Fig. 7E, 7E’), suggesting a regeneration defect. This defect was dose-dependent, with a higher NZ dose causing a much thinner OE and a much thinner OMP+ mature OSN zone (Fig. 7E, 7E’). By Day 28, the OE thickness and the OMP+ mature OSN zone thickness were further recovered in both non-NZ-treated control and NZ-treated mice. In NZ-treated mice, the OE thickness and the OMP+ mature OSN zone thickness were still thinner (Fig. 7E, 7E’). These results suggest that the loss of asymmetric HBC division leads to impaired OE regeneration.

To evaluate the functional impact of disrupted asymmetric HBC division on OE regeneration, we conducted a buried food-seeking behavioral test on NZ-treated mice at Day 15 and 28 post-injury. At Day 15, 75% of control mice located the buried food pellet within 10 minutes, whereas only 37% of NZ-treated mice succeeded (Fig. 7F). By Day 28, all control mice located the food in a timely manner, but 22% of NZ-treated mice still failed. These deficits in olfactory behavior align with the impaired OE regeneration in NZ-treated mice.

Together, these *in vivo* results support that asymmetric HBC division and asymmetric histone inheritance are required for the rapid regeneration of injured OE tissue and the timely recovery of olfactory function (Fig. 7G).

## DISCUSSION

In this study, we investigate the division modes and histone inheritance patterns of olfactory HBCs, a *bona fide* adult stem cell population, in the mouse olfactory epithelium. We have documented a substantial percentage of asymmetrically dividing HBCs, characterized by the asymmetric distribution of the ‘stemness’ transcription factor p63. For the first time in mammalian adult stem cells *in vivo*, we report asymmetric histone inheritance in asymmetrically dividing HBCs during early tissue regeneration following injury. Furthermore, asymmetric HBC division and the associated asymmetric histone inheritance correlate with asynchronous transcription re-initiation, observed in telophase HBCs both *in vivo* and in primary cultured HBCs. Culturing primary HBCs enables live-cell tracking and imaging, as well as isolation of paired HBC daughter cells for single-cell transcriptomic profiling. This transcriptomic analysis reveals priming for distinct cell fates after a single division in a substantial population of HBCs. To explore the biological relevance of asymmetric histone inheritance, we used the microtubule depolymerization drug NZ to disrupt histone asymmetry, which resulted in the loss of both asymmetric p63 distribution and asynchronous transcription re-initiation. NZ treatment in injured animals significantly impaired olfactory sensory neuron regeneration and olfactory behaviors, underscoring the importance of asymmetric histone inheritance in tissue repair and regeneration. Overall, this study provides new insights into the molecular mechanisms, cellular dynamics, and biological significance of asymmetric histone inheritance and cell fate determination in mammalian tissue regeneration.

### Conservation and Nuances of Asymmetric Histone Inheritance

In *Drosophila* male germline and female intestinal stem cells, asymmetric histone inheritance has been shown to regulate the distinct cell fates in asymmetrically dividing stem cells, suggesting a common mechanism across different adult stem cell systems in this invertebrate model organism^53,65^. However, evidence for asymmetric histone inheritance in other organisms, including mammals, has been lacking. In this study, we report the first direct evidence of asymmetric histone inheritance in a mammalian adult stem cell lineage, the mouse olfactory HBCs.

We found that histone level asymmetry in olfactory HBCs is more pronounced for canonical histones H3 and H4 but less so for H2A and H2B, consistent with previous findings in *Drosophila* adult stem cells. However, the non-canonical histone variant H3.3, whose incorporation is independent of DNA replication but relies on transcriptional activity^66,67^, also shows asymmetric inheritance. This finding sharply contrasts with the symmetric H3.3 inheritance observed in *Drosophila*. These data suggest that histone tetramer (H3/H3.3-H4)₂ act as key carriers of epigenetic information in olfactory HBCs. The commonalities and differences between the mouse HBC system and *Drosophila* adult stem cell lineages may arise from species-specific factors, cell type differences, and/or interactions with other epigenetic mechanisms. For instance, while DNA methylation plays a crucial role in the mammalian epigenome, it is almost negligible in adult flies^68–71^. Further studies are required to fully understand the biological implications of these differences and the underlying mechanisms.

Histone composition and distribution are tightly associated with higher-order chromatin organization, which allows for the differential binding of RNA polymerase and transcription factors, thereby regulating gene expression. We found that RNA Polymerase II and the key transcription factor p63 were differentially associated with the two sets of sister chromatids, indicating differences in chromatin structure underlies asynchronous transcription re-initiation and rebinding of transcription factors during late telophase in olfactory HBCs. Consistent with the asymmetric distribution of the transcription-dependent histone variant H3.3, these findings reveal that differential transcription re-initiation in daughter cells represents a key biological consequence of asymmetric histone inheritance. This finding complements previous observations of asynchronous DNA replication initiation in the daughter cells of *Drosophila* male germline stem cells^56^.

Previous studies have shown that asymmetric mitotic spindle activity is required for partitioning histone asymmetrically in *Drosophila* male germline stem cells^29,72^. Live-cell imaging revealed asymmetric microtubule activity in dividing primary cultured HBCs. Consistent with findings in *Drosophila*, the microtubule depolymerization drug NZ disrupted asymmetric microtubule activity, resulting in the loss of asymmetry in histone inheritance, p63 association, and RNA Pol II phosphorylation in HBCs.

In summary, our study has revealed the conserved features and system-specific nuances of asymmetric histone inheritance across adult stem cell systems, ranging from *Drosophila* to mice and involving tissue homeostasis and regeneration. The shared and distinct mechanisms include *cis*-elements incorporated in chromosomes, such as histones and histone variants, *trans*-factors, such as microtubules and transcription factors that interact with chromosomes, and biological outcomes, such as cell cycle progression and transcription re-initiation. These findings underscore the importance of studying asymmetric histone inheritance in a context-dependent manner. Furthermore, given the high conservation of histone proteins and their functions, asymmetric inheritance of histones and/or histone variants holds promise as a marker for identifying and tracing stem cell activities and asymmetric cell division across diverse systems and organisms *in vivo*.

### Revisiting the Mitotic Gene “Bookmarking” Phenomenon

In multicellular organisms, cell fate is determined by the unique gene expression profile of each cell type, dictated by its transcriptome. During mitosis, transcription activity is globally silenced as chromatin becomes highly condensed, restricting access to the transcription initiation complex and transcription factors. However, a subset of transcriptional factors has been found to remain bound to their genomic targets throughout mitosis-a process known as mitotic gene bookmarking, which helps preserve transcriptional memory^73^.

To understand how two daughter cells inheriting identical genetic codes can adopt distinct fates following ACD, it is crucial to investigate the mechanisms that reactivate transcription upon mitotic exit. Studies in cultured cells have primarily focused on how the interphase transcriptome resumes post-mitotically to maintain cell fate consistently^74,75^. However, how transcription programs are asymmetrically re-established after ACD to drive cell fate changes remains largely unexplored, representing a crucial question in understanding development, tissue homeostasis and regeneration across tissues and species.

We found that epigenetic differences between daughter cells, particularly in histones and histone variants, contribute to differential transcription re-initiation, transcription factor re-binding, and the adoption of distinct cell fates during ACD of HBCs. Spatially, transcriptional activity strongly correlates with the enrichment of the key ‘stemness’ transcription factor p63. Using antibodies against RNA Polymerase II phosphorylated at Ser5 (initiating Pol II) and Ser2 (elongating Pol II), we discovered asymmetric active transcription linked to asymmetric histones on the segregated sister chromatids. This finding was further validated using the uridine analog-EU as a marker for newly transcribed RNAs. Temporally, as chromosomes condense from prophase to metaphase, markers of active transcription and p63 are excluded from the condensed chromosomes. These markers asymmetrically re-associate with decondensing sister chromatids during anaphase and telophase as well as post-mitotic daughter nuclei.

Mitotic gene ‘bookmarking’ has been reported as a molecular mechanism to restore similar gene expression patterns after mitosis^76–81^, though caution is warranted when interpreting results from experiments involving cross-linking^82^. Our findings indicate a mechanism specific to adult stem cells during OE tissue regeneration. Instead of remaining bound to chromosomes throughout mitosis, the crucial ‘stemness’ transcription factor p63 dissociates from chromosomes during prophase to metaphase but dynamically re-associates asymmetrically with telophase chromosomes. This re-association corresponds to the chromatin status of the sister chromatids. For example, p63 enrichment at telophase chromosomes strongly colocalizes with the transcriptionally active H3.3 variant but shows distinct patterns from canonical histones H3, H2A and H2B, suggesting preferential enrichment at genomic regions with low nucleosome density. These results suggest that cell fate determination in ACD is initiated by intrinsic mechanisms, detectable as early as anaphase and telophase, rather than relying solely on extrinsic signals in the post-mitotic environment. This dynamic re-association of p63 provides new insights into how transcription factors contribute to lineage specification during tissue regeneration.

### Asymmetric Histone Inheritance in Mammalian Tissue Repair and Regeneration

In adult tissues, many types of stem cells divide asymmetrically to balance the stem cell pool with the differentiating cell population^1^. In this study, we started to understand the biological significance of ACD and asymmetric histone inheritance in mouse OE during tissue regeneration. In NZ disruption assay, asymmetrically dividing cells with asymmetric histone inheritance are lost, accompanied by delayed OE regeneration and defective olfactory behavior. These findings are in line with the previous report in *Drosophila* that epigenetic inheritance disruption led to defects in stem cell lineages, leading to phenotypes such as tumors, stem cell loss or stem cell hyperplasis^53,83^. One explanation for these results in the OE is that disruption of asymmetric histone inheritance altered chromatin architectures for differentially recruiting other *trans*-factors such as p63, leading to confused cell state and defective differentiation during OE repair. This raised the hypothesis that asymmetric histone inheritance may play important roles in regulating proper chromatin organization for asynchronous transcription initiation and proper gene expression. Further studies are needed to focus on the crosstalk between epigenetic inheritance, chromatin architecture, stem cell fate decisions in the *in vivo* tissues.

Along with intrinsic regulatory factors, the stem cell microenvironment, or ‘niche’, plays a critical role in maintaining stem cell functions^84^. Stem cell niches can be broadly classified into two types: stromal niches, such as the *Drosophila* germline stem cell niche, and epidermal niche, including the *Drosophila* intestinal stem cell niche and the olfactory epithelial niche related to this study^87^. In the epidermal niche, both asymmetric and symmetric cell divisions occur, as reported for *Drosophila* intestinal stem cells^53^. Here, both division modes were observed *in vivo* (Fig. 1C, 1C’) and in primary cultured cells (Fig. 4C–F). However, the mechanisms regulating these division modes in HBCs remain unclear and require further investigation. This study provides a powerful platform to investigate the niche regulation of adult tissue stem cells. Specifically, the identification of asymmetrically dividing cells with asymmetric histone distribution can help to more accurately identify different subtypes of *bona fide* adult stem cells and locate their niche factors across different tissue and organisms, which will greatly facilitate future studies on how adult stem cells interact with the niche factors to modulate their cell fates in both physiological and pathological conditions.

In summary, our results suggest that the asymmetric histone inheritance may function to regulate differential transcription re-initiation and determine distinct cell fates in mouse olfactory tissue regeneration. Those studies provide insights into the biological significance of epigenetic histone inheritance, which will help understanding the mechanisms of how epigenetic factors contribute to adult tissue repair and regeneration.

## METHODS

### Mouse Lines

All experiments were performed on adult animals at 6–8 weeks of age. Female and male data were both used for cell culture and behavior analysis. The mice were on a mixed C57BL/6 × 129 (B6;129) genetic background and maintained with open access to food and water under a 12-hour light/12-hour dark cycle. TRE3G-H4-mScarlet and TRE3G-H3.3-mScarlet mouse strains were obtained from Sue Hammoud laboratory, p63-EGFP reporter mouse strain was from Sinha laboratory, and B6N.FVB(Cg)-Tg(CAG-rtTA3)4288Slowe/J mouse strain was form Jackson lab (Strain #:016532). All animal experiments were conducted according to the procedures approved by the Johns Hopkins University IACUC guidelines (approval protocol numbers MO19A127/MO22A71/MO24A114).

### OE Cryosection Preparation

Mice were sacrificed following methimazole (50 mg/kg) i.p. injection. Half heads, with an intact olfactory epithelium, were collected for cryosection. For cryopreservation, samples were first washed in PBS, then treated with 4% PFA overnight. The sample was then treated with 0.5 M EDTA for 1 day, 10% sucrose overnight, and 30% sucrose overnight at 4 °C. The sample was then embedded in O.C.T and stored at −20° C and cut into 8–10 μm slices onto Fisherbrand^™^ Superfrost^™^ Plus Microscope Slides (12–550-15, Fisher Scientific) via cryosection.

### Primary HBC Culture with Extracellular Matrix

The primary HBC culture was performed based on the previously published method^1^ with optimized culture conditions. Briefly, OE tissues were dissected from adult mice (6–8 weeks old) 2 days after the methimazole injection. The collected OE tissue was finely minced in the complete HBC culture medium and then treated with 1 × collagenase/hyaluronidase at 37°C for 45 mins. Samples were centrifuged at 1,200 × *g* for 5 minutes, and the cell pellets were resuspended in 5 mL pre-warmed 0.25% TrypLE (12604013, Thermo Fisher) at 37°C for 2 minutes followed by addition of 10 mL of ice-cold PBS without calcium or magnesium. The samples were then centrifuged at 1,200 × *g* for 5 minutes, resuspended in 5 mL of pre-warmed dispase (07923, Stemcell Tech) and 10 μL of DNase 1 (07900, Stemcell Tech) and briefly triturated at 37°C for 1 min. Then, 10 mL of ice-cold PBS without calcium or magnesium were added and samples were filtered through Falcon 40 μm cell strainers into 50 mL conical centrifuge tubes. The cells were collected and cultured in the complete HBC medium. To maintain HBCs in different activation status, we employed two types of extracellular matrix, Tropoelastin and Fibronectin. Before seeding the cells, the culture dishes were coated with Tropoelastin or Febronectin overnight.

### Live Cell Imaging of HBCs

The cultured HBCs were infected with lentivirus (Lenti-CAG-H4-dendra2 or Lenti-CAG-FUCCI-CA5, from HHMI core facility) at ~10 MOI in the HBC medium for 1 day and then maintained in fresh HBC medium for 1 more day to allow for fluorescent reporter expression. Live cell imaging of HBCs was performed on STELLARIS 5 confocal microscope equipped with a Leica HC PL APO 63× 1.40-NA oil CS2 objective, a white light laser and power HyD S detector. The objective was maintained at 37°C throughout the experiment by objective heater. 5% CO2 and 100% humidity were maintained by Tokai hit STX stage top incubation system. The same imaging procedure was followed for imaging of the live tubulin (SPY555-tubulin) and live DNA (SPY555-DNA) dye.

### Immunofluorescence Staining

The slides with OE tissue sections were boiled in 0.01 M citrate buffer (pH 6.0) for 20 mins for antigen retrieval in a pre-heat electric steamer. Then treated with 0.5% Triton X-100 in PBS (PBST) with primary antibodies diluted in 5% BSA in PBST, followed by detection with Alexa secondary antibodies (Invitrogen) as described^2^. The primary antibodies and dilutions used were as follows: mouse monoclonal p63, 1:100 (D-9, sc-25268; Santa Cruz); rabbit monoclonal Keratin5, 1:500 (NBP3–15374, NOVUS); rabbit monoclonal Ki67, 1:250 (NB600–1252, NOVUS); rabbit polyclonal RNA polymerase II RPB1 phospho S2, 1: 800 (AB5095, Abcam); rabbit polyclonal RNA polymerase II RPB1 phospho S5, 1: 800 (AB5131, Abcam); mouse monoclonal alpha Tubulin (DM1A), 1:500 (14–4502-82, Thermo Fisher); rabbit monoclonal Histone H4 (AF5215-SP, Novus); rabbit monoclonal Histone H3 (A17562, ABclonal); rabbit monoclonal Histone H3.3, 1:250 (NBP2–67530, NOVUS); ChromoTek Histone-Label Atto488, 1:400 (tba488, Proteintech); rabbit monoclonal anti-phospho-Histone H3Thr3 (JY325), 1:800 (05–746R, Millipore Sigma); Goat anti-Olfactory Marker Protein (OMP), 1:500 (544–10001, WAKO). Nuclei were counterstained using Hoechst 33342 and slides were coverslipped with VectaShield mounting medium (Vector Laboratories, Inc.).

### EU Pulse-Labeling in Primary Culture HBCs

Primary cultured HBCs were labelled with 0.5 mM 5-ethynyluridine (EU) in complete HBCs medium for 1 hour at 37°C. Then the media with EU was removed and the cells were washed three times with PBS and fixed with 4% PFA at 4°C overnight. To visualize the EU, HBCs with primary antibodies staining were subsequently processed for the click reaction with Alexa A555-azide (ThermoFisher, A20012) and then incubated with secondary antibodies.

### EdU Pulse-Labeling of Mouse OE Tissue

EdU labeling was incorporated using the Click-iT Plus EdU Alexa Fluor 647 Imaging Kit (Invitrogen C10640) or Click-iT Plus EdU Alexa Fluor 594 Imaging Kit (Invitrogen C10339) according to manufacturer’s instructions. Prepare the EdU stock at the concentration of 2.5 mg/mL. 6–8-week-old mice were injected i.p. with 100 μL dissolved EdU in PBS and the OE tissues were harvested 2 hours after EdU injection. The samples were later fixed and cryo-sectioned, as described in the cryosection preparation and immunostaining procedures above. Fluorophore conjugation to EdU was performed according to the manufacturer’s instructions, prior to secondary antibody incubation.

### Imaging Acquisition

Samples were imaged under a LSM700 confocal microscope or STELLARIS 5 confocal microscope equipped with a Leica HC PL APO 63× 1.40-NA oil CS2 objective, a white light laser and power HyD S detector. Z-stacks of 0.5 μm per layer were taken for mitotic HBCs. The ImageJ software was used to quantify the fluorescent intensities and evaluate the division angle of anaphase and telophase HBCs.

### The 3D Quantification of Telophase HBCs

To quantify the total amount of proteins (such as histones, p63 and RNA Pol II), we conducted a 3D quantification in volume by measuring the fluorescent signal in each plane from the Z-stack^3,4^ (as described in Figure S1C). Specifically, the 3D quantification of the fluorescent signal was done manually using ImageJ. Un-deconvolved raw images as 2D Z-stacks were saved as un-scaled 16-bit TIF images, and the sum of the gray values of pixels in the image (“RawIntDen”) was determined using Image J. The gray values of the fluorescent signal pixels for each Z-stack were calculated by subtracting the gray values of the background signal pixels from the gray values of the raw signal pixels. The total amount of the fluorescent signal in the nuclei was calculated by adding the gray values of the fluorescent signal from all Z-stacks. The total amount of fluorescent signals would represent the total amount of protein in the nuclei and be used for quantifying the asymmetric and symmetric protein distribution in telophase HBCs.

### The Division Angle Measurement

To measure a division angle of HBCs, use the ImageJ - “Angle” tool to click on three points that define the angle you want to measure. As shown in Figure 1D, the olfactory lamina propria was used to define the basal layer, and the division axis was established by the two sets of sister chromatids. During analysis, the division angles of anaphase and telophase HBCs at 60°−90° to the basal layer were classed as perpendicular; those that were at 0°−30° were classed as parallel. The perpendicular and parallel divisions were used for the quantification in Figure 1E’.

### Co-localization Analysis

The co-localization assay was performed using FIJI (ImageJ) software. The image was imported into ImageJ, and the HBCs identified via P63 fluorescent signals. Drawing near the edge of the cell to the best of our ability, we outlined the HBCs of interest using the “freehand selections” tool on the toolbar. After outlining the cell, the selected area was duplicated using a function under the toolbar (Image>Duplicate Image). The duplicated area is then split into individual channels using the toolbar (Image>Color>Split Channels). With this, the images are ready for the co-localization analysis using the Coloc 2 plugin (Analysis>Co-localization>Coloc 2). The Coloc 2 tool implements and performs the pixel intensity correlation over space (pixel intensity spatial correlation analysis). After opening the Coloc 2 plugin pop-up, select the two desired channels to perform the analysis on into “Channel 1” and “Channel 2.” We used Spearman’s Rank Correlation value to compare co-localization between different datasets. The result is +1 for perfect correlation, 0 for no correlation, and −1 for perfect anti-correlation. The co-localization analysis was performed between p63 and different histones (H4, H2A-2B, H3.3 and H3).

### Buried Food Seeking Behavior Test

The 6–8-week-old C57BL/6J wild-type mice of both sexes were used for the NZ treatment and food seeking behavior test. Before the behavior test, all the mice were deprived of food for 24 hours but with an open water supply. The animals were habituated in the test room for 30 minutes before the test. ~0.5 g peanut bar was buried at a random location underneath ~1.5 cm of fresh bedding in a clean mouse cage (46 cm L × 23.5 cm W). If an animal found the peanut bar in less than 10 min, it was counted as a success and the time spent finding the peanut bar was recorded. If an animal failed to find the peanut bar in 10 min, the test is terminated.

### Western Blot Analysis

Proteins were extracted from one side of OE tissue using 150 μL of RIPA lysis buffer containing Protease Inhibitor (P8340, Sigma). The OE tissue sample was physically disrupted by electronical homogenizer and then followed by an ultrasonication before centrifugation at ~14,000 g at 4 ℃ for 10 min. The supernatant was collected and boiled in 1×SDS loading buffer at 95 ℃ for 10 min. Western blot analysis was performed as previously described^2^. The proteins were separated by 4–12% Novex Tris-Glycine mini protein gel (XP04120BOX, Thermo Fisher), and then transferred to nitrocellulose membranes (Life Sciences). After incubating with StartingBlock^™^ Blocking Buffer (37543, Thermo Fisher) for 1 h at room temperature, the membranes were incubated overnight with histone H4 antibodies (1:1000, Abcam, ab31830) at 4 ℃, then incubated at room temperature with horseradish peroxidase-conjugated (HRP)-conjugated secondary antibodies (1:1000, CST, 7074S) for 1 h at room temperature. The membranes were developed using SuperSignal^™^ West Pico PLUS Chemiluminescent Substrate kit (Cat# 34577, Thermo Fisher). The immunoreactive protein bands were visualized using a G:Box chemi XRQ gel doc system (Syngene).

### Single Cell RNA-seq and Data Analysis

The single cell RNA-seq (scRNA-seq) experiment was done following the G&T-seq protocol^5^. Single daughter cells after division of ex vivo cultured primary HBC cells were collected individually using mouth pipette with pairing information noted. 1 ml of pre-diluted (1:10^6^) ERCC spike-in (Invitrogen Cat# 4456740) was added to the single cell lysate. RNA in the lysate was captured by oligo-dT beads and subjected to reverse transcription. cDNA from a single cell was amplified by 18 PCR cycles before dual indexing with the Nextera XT kit (Illumina Cat# FC-131–1096). The quality of single cell libraries was confirmed by TapeStation and libraries of 96 cells from 48 HBC pairs were pooled together for sequencing (150 bp paired-end) in one lane on the Illumina NovaSeq X Plus platform (Novogene US).

The quality of all FASTQ files from Illumina sequencing was analyzed and confirmed by FastQC (v0.12.1). scRNA-seq reads were trimmed with Trimmomatic (v0.39)^6^. The trimmed RNA-seq reads with both pair mates were aligned by STAR (v2.7.11a)^7^ to the GRCm38 annotation (ENSEMBL release 100) plus ERCC information. StringTie (v2.2.1)^8^ was used to generate counts of genes in the GTF reference. Publicly available single-cell RNA-seq datasets of *in vivo* retrieved HBC cells were downloaded from Gene Expression Omnibus (accession numbers: GSE99251, GSE95601)^9,10^. scRNA-seq count matrices of all single cells were analyzed by Seurat (v5.1.0)^11^. The “CCAIntegration” method in Seurat was used when integrating the published scRNA-seq data into the newly generated HBC pair datasets before visualization in UMAP. The newly-generated scRNA-seq dataset in this study was deposited to Gene Expression Omnibus (accession numbers: GSE286046).

After clustering of the single cells from HBC pairs by Seurat, the RNA-seq results of all cells falling into the “Activated HBC” cluster were treated as one group and compared to data of cells from the “Renewed HBC” cluster using DESeq2 (v1.44.0)^12^. ERCC spike-in was used for normalization. Log_2_ fold change = ±1 with adjusted R value < 0.1 was used as cutoff for significantly up- or down-regulated genes. Significantly upregulated genes were then used for gene ontology analysis with DAVID^13^. ERCC normalized gene counts were used for violin plots, and R values were calculated by a two-tailed t test.

### Mathematical Modeling Simulations

#### Mathematical model of cell cycle and HBCs activation

We consider a model based on ordinary differential equations to capture OE tissue regeneration dynamics after tissue injury. At an intuitive level, the model works by considering the activation of otherwise quiescent HBCs after injury and activated HBCs undergo asymmetric division leading to the formation of differentiated cells. The post-injury buildup of differentiated cells reduces HBC proliferation via feedback regulation, and HBCs return to their original quiescent state upon OE regeneration ensuring tissue homeostasis. We next describe the mathematical model in detail.

Let $H_{1},\;H_{2}$, and $H_{3}$ denote the fraction of HBCs that are in the G1, S, and G2/M phases of their cell cycle at time t after tissue injury. The transition from G1 to S is assumed to occur with rate $k_{1}$. As we will soon see, this rate $k_{1}$ is assumed to depend on a feedback signal from differential cells, such that the absence of differentiated cells triggers HBC activation by enhancing this rate and reducing the G1 phase duration. The transition from S to G2/M occurs with rate $k_{2}$ and we set this rate to be *k*_2_ = 1.33 *day*^−1^ that corresponds to the duration of S phase to be 1/*k*_2_ ≈18 hours consistent with single-cell microscopy data. Cells in the G2/M undergo division with rate *k*_3_ = 6 *day*^−1^ that corresponds to the average time spent in the G2/M phase to be 4 hours. The division of HBCs results in the formation of another HBC in G1 phase, and a differentiated cell whose population number we denote by D. This model results in the following system of differential equations that predict the temporal dynamics of $H_{1},H_{2},H_{3}$ and D:

$$\frac{dH_{1}}{dt}\;=-k_{1}H_{1}+k_{3}H_{3}$$

$$\frac{dH_{2}}{dt}=k_{1}H_{1}-k_{2}H_{2}$$

$$\frac{dH_{3}}{dt}=k_{2}H_{2}-k_{3}H_{3}$$

$$\frac{dD}{dt}=k_{3}H_{3}-\gamma D$$

where γ is the turnover rate of differential cells. As mentioned earlier, tissue homeostasis is feedback regulated by making the HBC G1 phase duration dependent on the differential cells via the Hill equation $k_{1}=\frac{k_{max}}{1+{\left(\frac{D}{K}\right)}^{h}}$ for some positive constants $k_{max},K$, and Hill coefficient h. The absence of differential cells D=0 shortens the G1 phase duration by increasing the rate at its maximum value $k_{1}=k_{max}$.

We use $H_{2}$, i.e., the fraction of HBCs in the S phase as the mathematical counterpart to the experimentally measured p63+/EdU+ fraction in Fig. 1B. In the absence of tissue injury, we see basal levels of HBC proliferation with the fraction of HBCs (p63+/EdU+) seen to be roughly 6.5% in control samples (Fig. 1C). Using this as an initial condition for the model together with D=0, we fit the model predicted dynamics of $H_{2}$ to data by minimizing the least square error between them. We further assume an additional parameter τ that represents an initial time-delay between tissue injury and HBC activation, with $H_{1},\;H_{2}$, and $H_{3}$ remaining at their basal levels for time t<τ and following the above differential equation for t>τ. The fit of $H_{2}$ to data is illustrated in Fig. 1B with feedback parameters estimated as $k_{max}=2.27{\;day}^{-1},\;\tau \;=\;0.8\;day\;,\;h\;=\;2\;,\;K\;=\;0.4\;a.\;u$. and $\gamma \;=\;0.06\;{\;day}^{-1}$.

#### Mathematical model of asymmetric cell division and transcription binding affinity

To model transcription re-initiation during mitotic exit, we analyze the RNA Pol II ratio between sister chromatids in HBCs during telophase. The histogram of the ratios in Figure S6A shows a bimodal distribution consistent with a fraction of cells undergoing symmetric cell division (i.e., equal partitioning of RNA Pol II between sister chromatids) vs. asymmetric cell division (i.e., preferential binding of RNA Pol II to one of the sister chromatids). We computed the statistics of this ratio measured across 55 single cells and obtain the mean, coefficient of variation, and skewness of the ratio to be 1.4 ± 0.08, 0.21 ± 0.03, 0.58 ± 0.45, respectively, where the ± denotes the 95% confidence interval as obtained by bootstrapping. We next develop a mathematical model to predict and match these statistics.

Let a cell undergo symmetric cell division with probability $p_{s}$ and RNA Pol II is partitioned symmetrically between the sister chromatids. In this case, we consider the RNA Pol II intensities $X_{1}$ and $X_{2}$ at the two sister chromatids to be drawn from a Gaussian distribution with a mean of one and standard deviation σ, where σ corresponds to the technical noise in RNA Pol II intensity quantification. In this case of symmetric division, the RNA Pol II intensities and their ratio r are determined as $X_{1}\;\sim \;N(1,\sigma ),\;X_{2}\;\sim \;N(1,\sigma ),r=\frac{Max\left(X_{1},X_{2}\right)}{Min\left(X_{1},X_{2}\right)}$. With probability $1-p_{s}$ a cell undergoes asymmetric division, where RNA Pol II is preferentially partitioned towards one sister chromatid and the RNA Pol II intensities $X_{1}$ and $X_{2}$ are determined by $X_{1}\;\sim \;N(1-\left.p_{b},\sigma \right),\;X_{2}\;\sim \;N\left(1+p_{b},\sigma \right),\;r=\frac{Max\left(X_{1},X_{2}\right)}{Min\left(X_{1},X_{2}\right)}$ where $p_{b}$ represents the bias in RNA Pol II preferentially binding to one sister chromatid over the other, with $p_{b}=0$ corresponding to symmetric RNA Pol II partitioning. In both cases the ratio r is computed by dividing the maximum of the two random variables $X_{1}$ and $X_{2}$ by their minimum.

The proposed model has three parameters: $p_{s}$ the probability of symmetric cell division, σ representing noise in RNA Pol II abundance quantification, and $p_{b}$ the bias in RNA Pol II binding for asymmetric cell division. For given values of $p_{s},\;\sigma ,\;p_{b}$ we simulated this model for 5,000 in-silico cells, and the statistics (mean, coefficient of variation, skewness) of the ratio r obtained from simulations were matched with corresponding statistics obtained from data (as reported above). This process results in the estimation of parameters as $p_{s}\;\approx \;0.45,\;\sigma \;\approx \;0.09,\;p_{b}\;\approx \;0.23$, and a sample histogram of ratios obtained from simulation is shown in Figure S6B These results imply an approximately 0.55 probability of asymmetric cell division, and in this case, the ratio of RNA Pol II binding affinity to one sister chromatid over the other is given by $\frac{1+p_{b}}{1-p_{b}}\approx 1.6$, implying a 60% increase in binding affinity.

### Statistical Analysis

Statistical evaluation was performed by Mann-Whitney unpaired t-test and Chi-Square test. Data are presented as Average ± SEM and significant difference between two groups were noted by asterisks: * p < 0.05, ** p < 0.01, *** p < 0.001, **** p< 0.0001. Statistical analysis was performed by GraphPad Prism 10 software. The fluorescence intensity was measured by Image J software.

## Supplementary Material

Supplement 1

## Data Availability

The scRNA-seq dataset in this study was deposited to Gene Expression Omnibus (GSE286046, tentative release date: Jan 07, 2027).
