# Supplementary material for: Asymmetric Histone Inheritance Regulates Differential Transcription Re-initiation and Cell Fate Decisions in Mouse Olfactory Horizontal Basal Cells
Source: bioRxiv. 2025 Mar 4:2025.03.02.641101. Preprint. [Version 1] doi: 10.1101/2025.03.02.641101 (PMC11908234; doi:10.1101/2025.03.02.641101)
Supplement: Supplement 1 [file NIHPP2025.03.02.641101v1-supplement-1.pdf]

bioRxiv preprint doi: https://doi.org/10.1101/2025.03.02.641101; this version posted March 4, 2025. The copyright holder for this preprint (which was not certified by peer review) is the author/funder, who has granted bioRxiv a license to display the preprint in perpetuity. It is made available under aCC-BY 4.0 International license.

SUPPLEMENTARY INFORMATION

Supplemental Figures and Figure Legends:

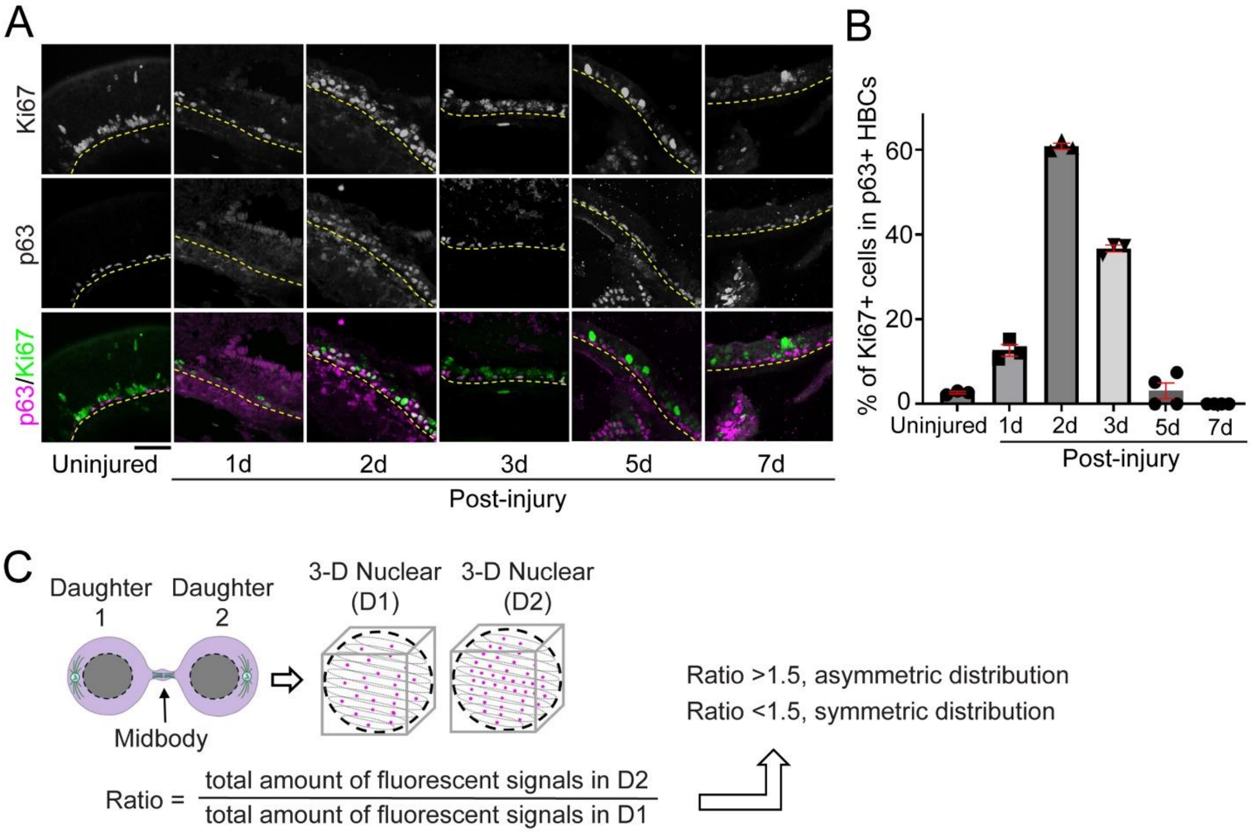

Figure S1: Post injury dynamics of Ki67 labeling and 3-D quantification in p63+ HBCs. (**A**) Post injury dynamics of Ki67 labeling in p63+ HBCs. Ki67 depicts cell proliferation activity. p63 (magenta) and Ki67 (green) were detected by immunostaining. Tissue sections from uninjured and injured olfactory epithelium at 1-, 2-, 3-, 5- and 7-days post-injury. Scale bar: 50 µm. (**B**) Ratio of Ki67+ cells in p63+ HBCs during early OE tissue regeneration. (**C**) Illustration of 3-D quantification of total amount of fluorescent signals and cutoff for asymmetric distribution in telophase HBCs.

bioRxiv preprint doi: https://doi.org/10.1101/2025.03.02.641101; this version posted March 4, 2025. The copyright holder for this preprint (which was not certified by peer review) is the author/funder, who has granted bioRxiv a license to display the preprint in perpetuity. It is made available under aCC-BY 4.0 International license.

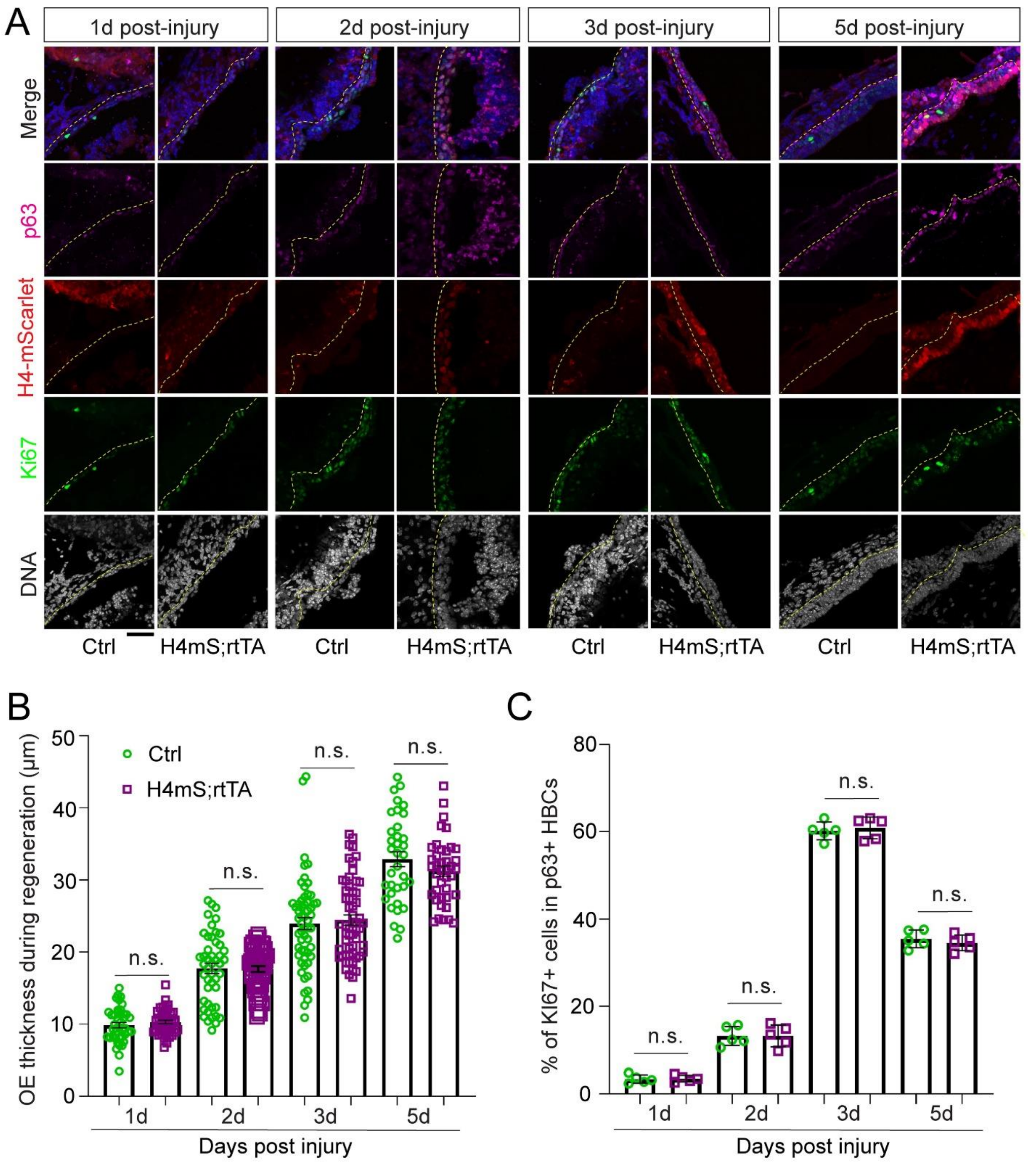

Figure S2: Normal early HBCs activation in H4mS;rtTA mice. (**A**) Dynamics of OE regeneration at 1d, 2d, 3d and 5d post-injury between H4mS;-rtTA control (Ctrl) and H4mS;+rtTA mice. Scale bar: 50 μm. (**B**) Qutification of OE thickness in H4mS;-rtTA control (Ctrl) and H4mS;+rtTA mice during regeneration. n.s., not significant by Mann Whitney test. (**C**) Percentage

bioRxiv preprint doi: https://doi.org/10.1101/2025.03.02.641101; this version posted March 4, 2025. The copyright holder for this preprint (which was not certified by peer review) is the author/funder, who has granted bioRxiv a license to display the preprint in perpetuity. It is made available under aCC-BY 4.0 International license.

of Ki67+ cells in p63+ HBCs in H4mS;-rtTA control (Ctrl) and H4mS;+rtTA mice during early OE regeneration. n.s., not significant by Mann Whitney test.

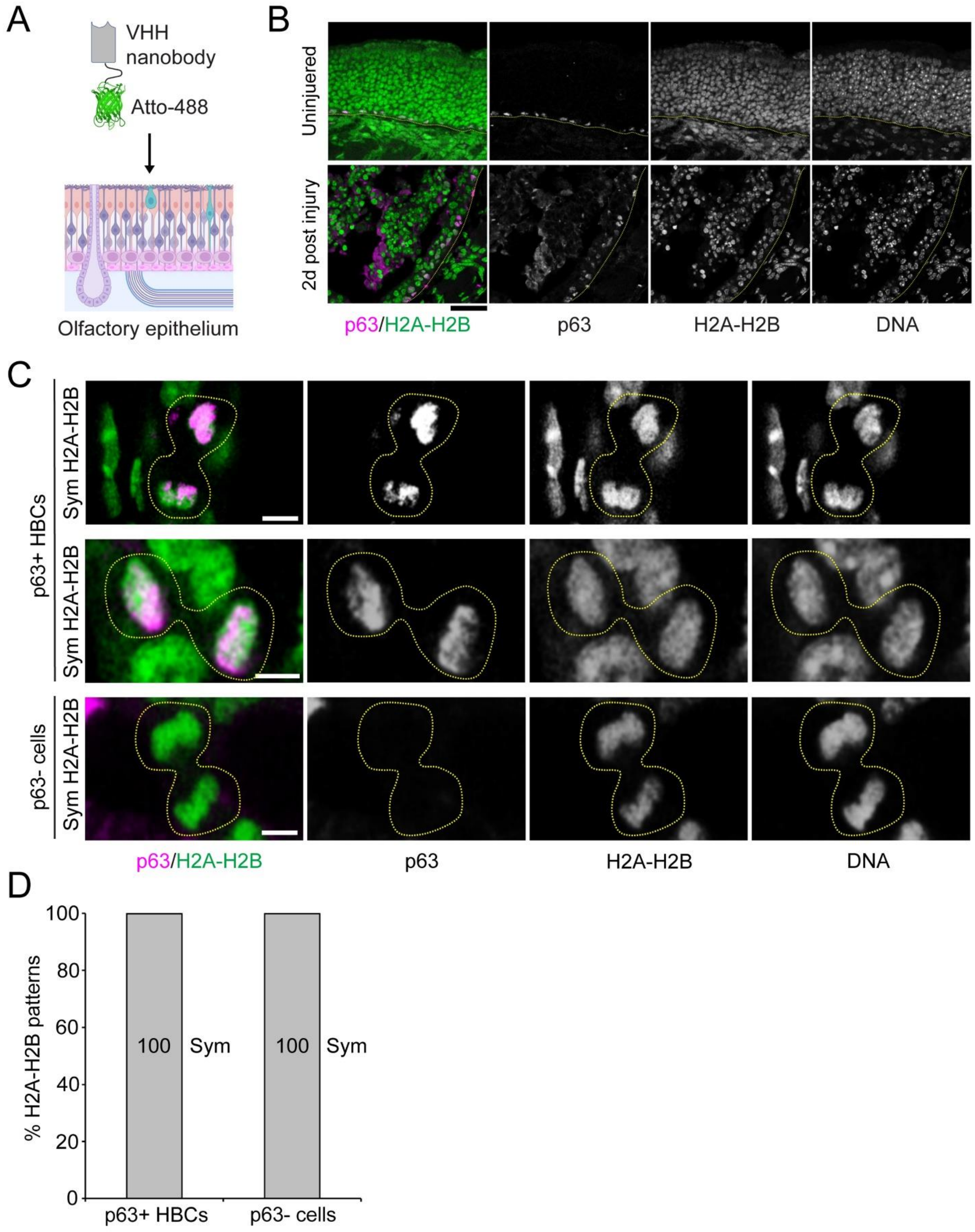

bioRxiv preprint doi: https://doi.org/10.1101/2025.03.02.641101; this version posted March 4, 2025. The copyright holder for this preprint (which was not certified by peer review) is the author/funder, who has granted bioRxiv a license to display the preprint in perpetuity. It is made available under aCC-BY 4.0 International license.

Figure S3: Symmetric histone H2A-H2B distribution in asymmetrically dividing HBCs. (**A**) Illustration of H2A-H2B nanobody staining in OE sections. Cartoon generated by Biorender.com. (**B**) Staining of H2A-H2B (green) and p63 (magenta) in uninjured and injured OE (2 days post-injury). (**C**) H2A-H2B distribution patterns in telophase HBCs. (top) a p63+ HBC with asymmetric p63 distribution and symmetric H2A-H2B distribution, (middle) a p63+ HBC with symmetric p63 distribution and symmetric H4 distribution, (bottom) a p63- HBC with symmetric H4 distribution, in OE section of wild type mice at 2 days post-injury. H2A-H2B (green), p63 (magenta) in merged panels. (**D**) Ratios of H2A-H2B distribution patterns in p63+ HBCs (N=20) and p63- cells (N=15) at telophase. Scale bar: 50 µm (**B**), 5 µm (**C**).

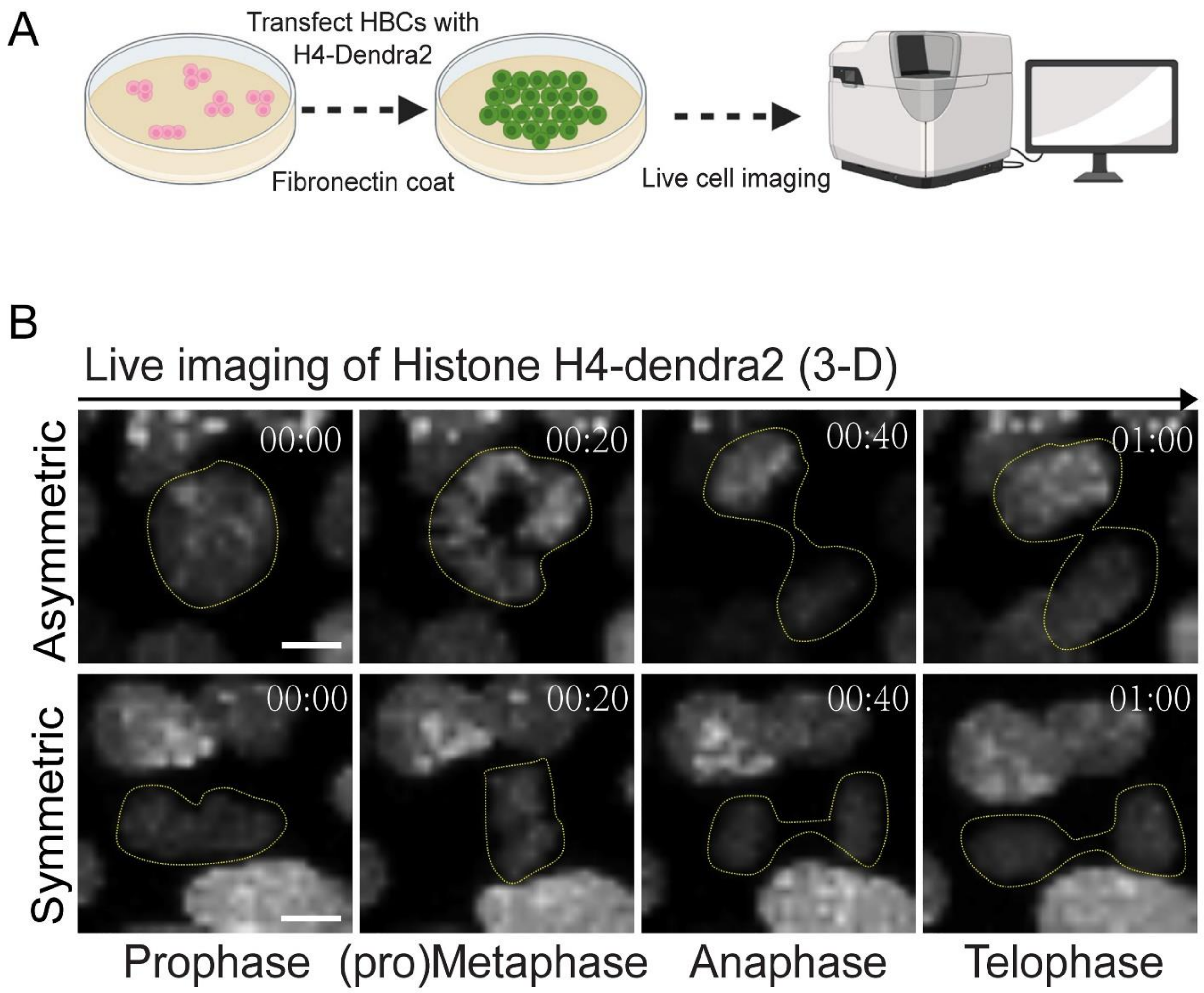

Figure S4: Live cell imaging shows asymmetric H4 segregation in primary culture HBCs. (**A**) Illustration of primary HBCs culture with Fibronectin coating conditions to drive stem cell

bioRxiv preprint doi: https://doi.org/10.1101/2025.03.02.641101; this version posted March 4, 2025. The copyright holder for this preprint (which was not certified by peer review) is the author/funder, who has granted bioRxiv a license to display the preprint in perpetuity. It is made available under aCC-BY 4.0 International license.

activation and study histone inheritance with live imaging of histone H4-Dendra2 transfected HBCs. (**B**) Live imaging of asymmetric and symmetric histone H4 segregation during mitosis of H4-Dendra2 transfected HBCs. Scale bar: 5 μm.

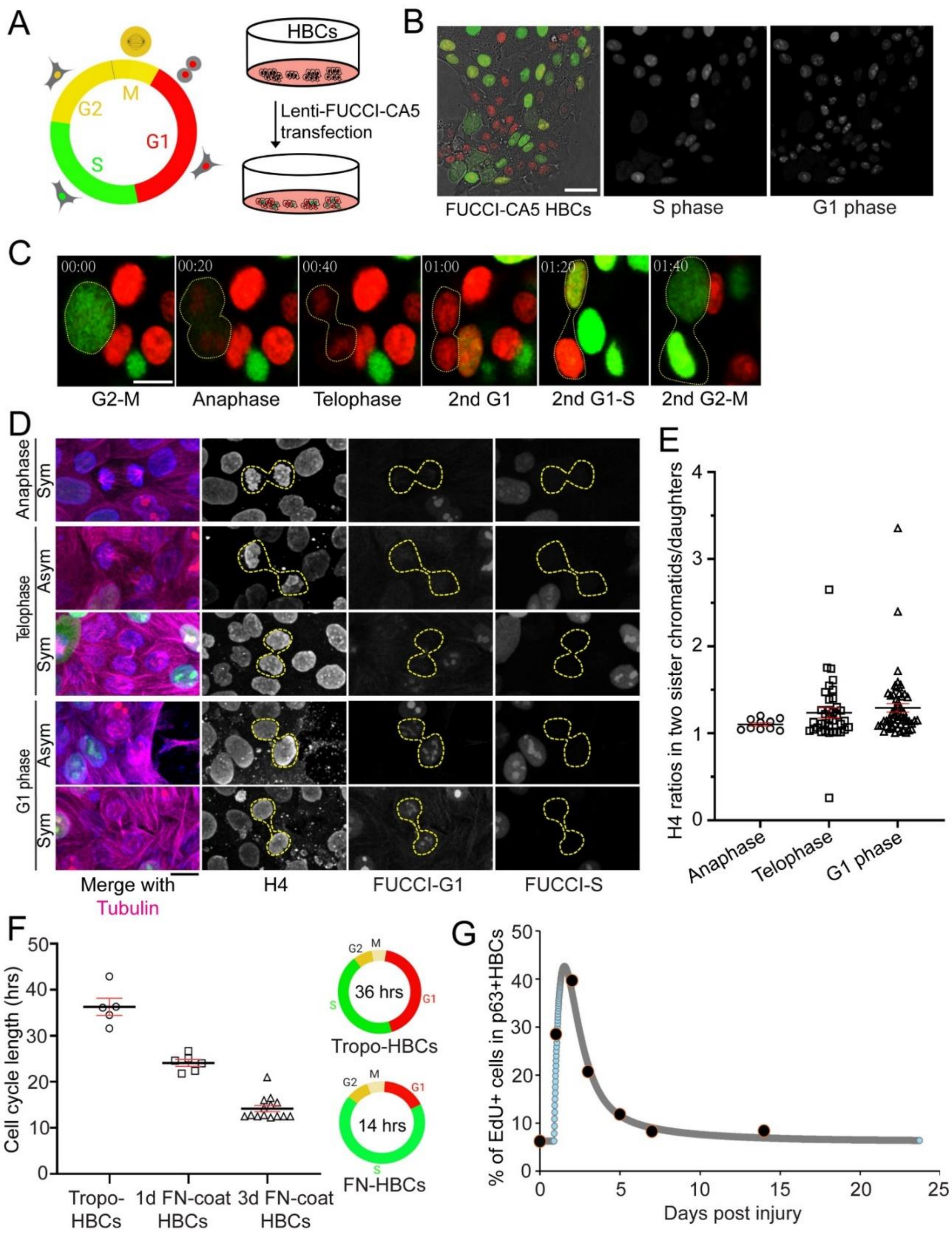

bioRxiv preprint doi: https://doi.org/10.1101/2025.03.02.641101; this version posted March 4, 2025. The copyright holder for this preprint (which was not certified by peer review) is the author/funder, who has granted bioRxiv a license to display the preprint in perpetuity. It is made available under aCC-BY 4.0 International license.

Figure S5: Asynchronized cell cycle progression in primary culture HBCs. (**A**) Illustration of FUCCI-CA5 reporter system and transfection of FUCCI-CA5 plasmid into primary culture HBCs to track cell cycle progression with live imaging. (**B**) Representative images of FUCCI-CA5 transfected HBCs with G1 phase cells (red) and S phase cells (green). Scale bar: 25 µm. (**C**) Live cell imaging of asynchronized G1-to-S phase transition with FUCCI-CA5 transfected HBCs. Scale bar: 10 µm. (**D**) Fixed cell images of histone H4 distribution (grey) with FUCCI reporter showing G1 phase (red) and S phase (green). Scale bar: 5 µm. (**E**) Quantification of H4 ratios in two sister chromatids of telophase HBCs and two daughter cells of G1 phase HBCs. (**F**) Cell cycle length of HBCs from Tropoelastin (Tropo) and Fibronectin (FN) coated conditions. (**G**) Mathematical modeling data of cell cycle changes and HBCs activation during OE regeneration.

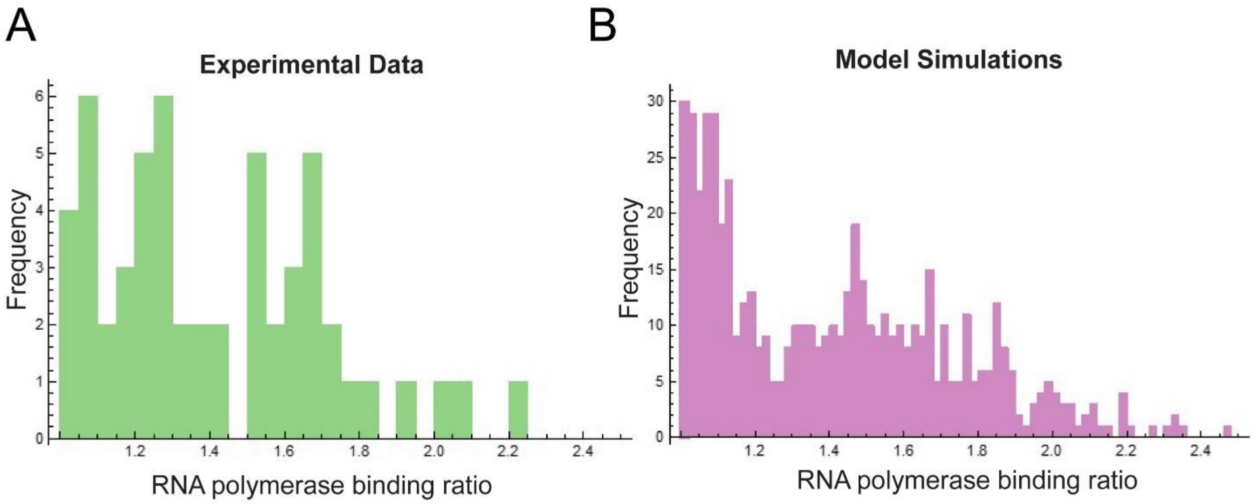

Figure S6: Mathematical modeling data of transcription re-initiation with differential binding affinity of RNA Pol II and asymmetric cell division in telophase HBCs. (**A**) Experimental data of RNA Pol IIS2ph distribution from telophase HBCs based on the immunostaining results. (**B**) Simulated modeling data of asymmetric cell division and RNA Pol IIS2ph transcription binding affinity.

bioRxiv preprint doi: https://doi.org/10.1101/2025.03.02.641101; this version posted March 4, 2025. The copyright holder for this preprint (which was not certified by peer review) is the author/funder, who has granted bioRxiv a license to display the preprint in perpetuity. It is made available under aCC-BY 4.0 International license.

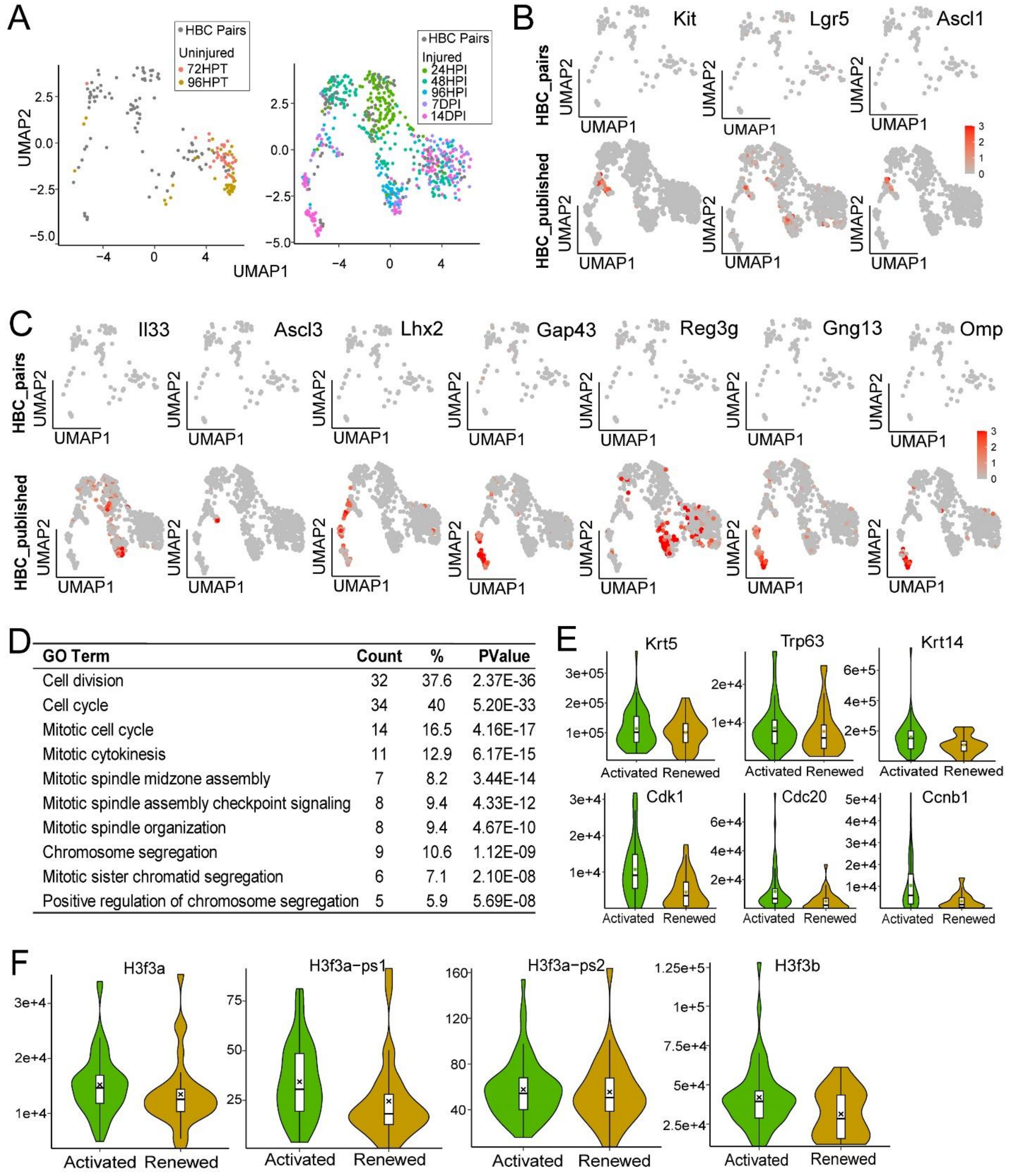

GO Term	Count	%	PValue
Cell division	32	37.6	2.37E-36
Cell cycle	34	40	5.20E-33
Mitotic cell cycle	14	16.5	4.16E-17
Mitotic cytokinesis	11	12.9	6.17E-15
Mitotic spindle midzone assembly	7	8.2	3.44E-14
Mitotic spindle assembly checkpoint signaling	8	9.4	4.33E-12
Mitotic spindle organization	8	9.4	4.67E-10
Chromosome segregation	9	10.6	1.12E-09
Mitotic sister chromatid segregation	6	7.1	2.10E-08
Positive regulation of chromosome segregation	5	5.9	5.69E-08

Figure S7: Analysis of scRNA-seq data of paired HBC daughters. (**A**) Integration of the newly generated scRNA-seq data using primary cultured HBC cells into published scRNA-seq results using *in-vivo* samples from uninjured OE (GSE99251) and injured OE (GSE95601). (**B**) Expression of GBCs marker genes *Ascl1*, *Kit* and *Lgr5* in HBC pairs and HBC published datasets.

bioRxiv preprint doi: https://doi.org/10.1101/2025.03.02.641101; this version posted March 4, 2025. The copyright holder for this preprint (which was not certified by peer review) is the author/funder, who has granted bioRxiv a license to display the preprint in perpetuity. It is made available under aCC-BY 4.0 International license.

(**C**) Expression of marker genes from different OE cell types, including sustentacular cell marker gene *Il33*; microvillous cell marker gene *Ascl3*; immediate neuronal precursor marker gene *Lhx2*; immature olfactory sensor neuron marker genes *Gap43*, *Reg3g* and mature olfactory sensor neuron marker genes *Gng13*, *Omp*. (**D**) Gene ontology analysis for biological processes related to genes highly expressed in activated HBC cells. (E) Expression of HBCs cell fate and proliferation associated genes in all samples. (**F**) Expression of genes controlling histone H3.3 in activated and renewed HBC cells.

bioRxiv preprint doi: https://doi.org/10.1101/2025.03.02.641101; this version posted March 4, 2025. The copyright holder for this preprint (which was not certified by peer review) is the author/funder, who has granted bioRxiv a license to display the preprint in perpetuity. It is made available under aCC-BY 4.0 International license.

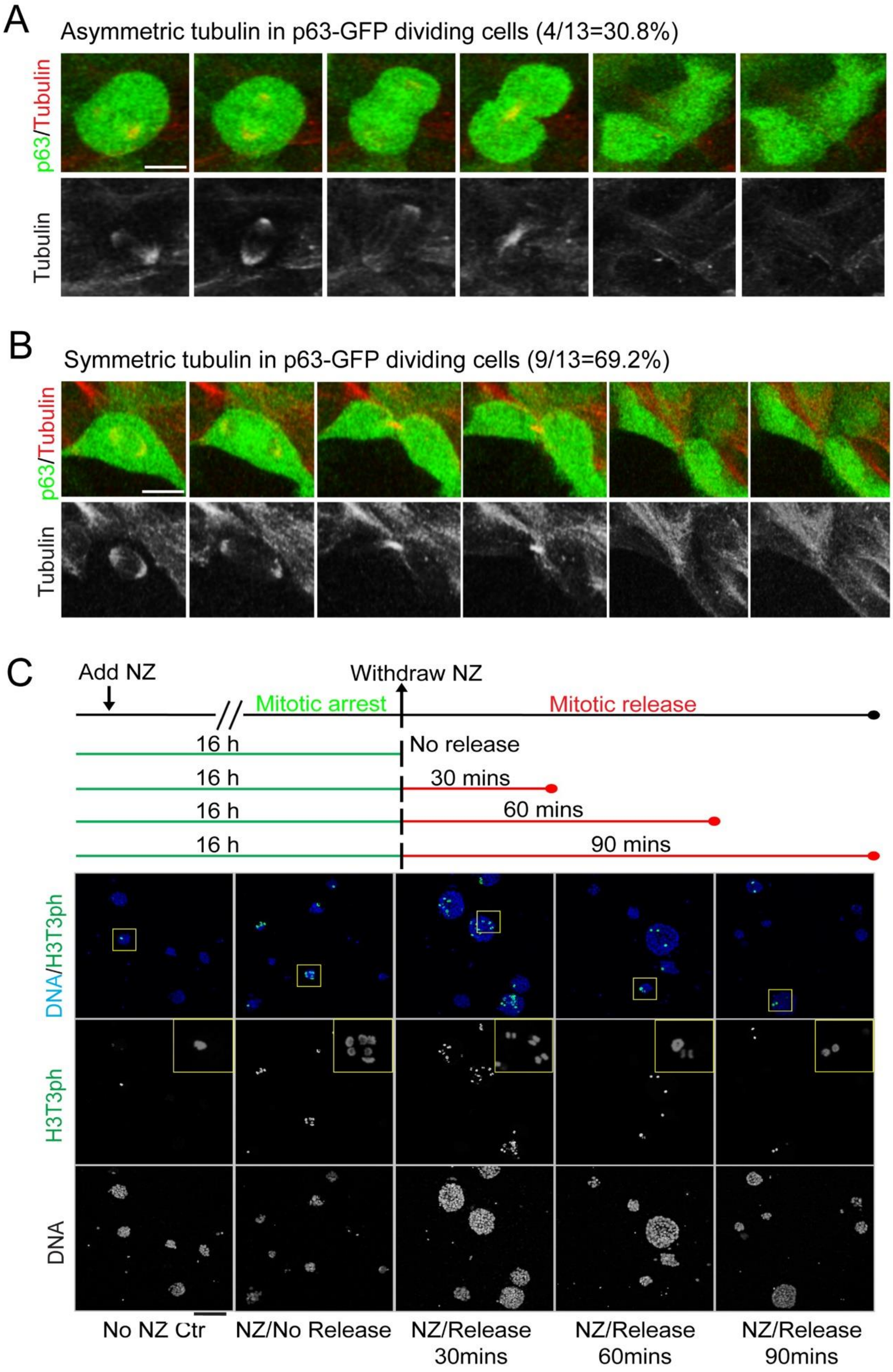

bioRxiv preprint doi: https://doi.org/10.1101/2025.03.02.641101; this version posted March 4, 2025. The copyright holder for this preprint (which was not certified by peer review) is the author/funder, who has granted bioRxiv a license to display the preprint in perpetuity. It is made available under aCC-BY 4.0 International license.

Figure S8: Asymmetric microtubule activity in asymmetrically dividing HBCs. (**A-B**) Live cell imaging of microtubule activity using live tubulin dye SiR-Tubulin (red) and *p63*-EGFP transcriptional reporter (green) during HBCs divisions (N=13 in total) with 20 minutes interval. (**A**) Asymmetric microtubule activity and different p63 transcript (N=4). (**B**) Symmetric microtubule activity and comparable p63 transcript (N=9). Scale bar: 5 µm. (**C**) Titration of Nocodazole (NZ) releasing time of synchronization treatments in the primary cultured HBCs. To access acute responses to NZ, we cultured HBCs with NZ for 16 hours, followed by release and imaging, After the 16-hour treatment, most HBCs were arrested at prometaphase. Upon release, HBCs re-entered mitosis in a time-dependent manner. The mitotic HBCs are indicated by H3T3ph (green) and DNA (blue). Scale bar: 100 µm.
